# It Is What It Isn’t: Introducing a Constraint-Based Approach to Structure Learning

**DOI:** 10.3390/e28050534

**Published:** 2026-05-07

**Authors:** Christoffer Lundbak Olesen, Nace Mikuš, Mads Hansen, Nicolas Legrand, Peter Thestrup Waade, Christoph Mathys

**Affiliations:** 1 Interacting Minds Centre, Aarhus University, Jens Chr. Skous Vej 4, 8000 Aarhus, Denmark; clo@cas.au.dk (C.L.O.); nace.mikus@cc.au.dk (N.M.); nicolas.legrand@cas.au.dk (N.L.); chmathys@cc.au.dk (C.M.); 2 Department of Philosophy, University of Bristol, Cotham House, Bristol BS6 6JL, UK; mads.hansen@bristol.ac.uk; 3 Translational Neuromodeling Unit, University of Zürich and ETH Zürich, Wilfriedstrasse 6, 8032 Zürich, Switzerland

**Keywords:** structure learning, constraint-based dynamics, cognitive modelling, delusions, hierarchical Gaussian filter, computational psychiatry

## Abstract

Biological cognition depends on learning-structured representations in ambiguous environments. Computational models of structure learning typically frame this as an inference problem, but often overlook the temporally extended dynamics that shape learning trajectories under ambiguity. In this paper, we reframe structure learning as an emergent consequence of constraint-based dynamics. Informed by the literature on the role of constraints in complex biological systems, we develop a constraint-based approach to computational cognitive modelling and provide a proof-of-concept model. The model consists of an ensemble of components, each comprising an individual learning process, whose internal updates are locally constrained by both external observations and system-level relational constraints. This is formalised using Bayesian probability as a description of constraint satisfaction rather than epistemic inference. Representational structure is not encoded directly in the model equations, but emerges over time through the interaction, stabilisation, and elimination of components under these constraints. Through a series of simulations in environments with varying degrees of ambiguity, we demonstrate that the model reliably differentiates the observation space into stable representational categories. We further analyse how global parameters controlling internal constraint and initial component precision shape learning trajectories and long-term behavioural alignment with the environment. We discuss the formal relationship between the present approach and Bayesian inference accounts, and argue that a constraint-based approach offers a conceptually distinct foundation for relating computational models to biological systems.

## 1. Introduction

Constraints are among the most fundamental organising principles of biological systems [1,2,3,4,5,6], from membranes enabling cellular homeostasis to skeletons enabling locomotion. Evidence suggests that the brain is an inherently active system, partially modulated by sensory input [7,8], which contrasts with a classic view of the brain as an input-driven system [9]. This primacy of internal activity over external activation indicates that the organising principles enabling cognitive capacities include the selective constraint of ongoing activity. Yet the sense in which constraints organise biological systems and enable their capacities are under-explored within the dominant frameworks for computational cognitive modelling.

Biological cognitive agents need to learn from undifferentiated observations, often while facing an ambiguous environment. To function effectively under such conditions, they must differentiate these observations to support adaptive behaviour. This requires the construction of an internal representational structure that determines what differentiable categories perceptually exist for the organism [10].

In this paper, we propose a constraint-based approach to computational modelling in which cognitive capacities are understood as emerging through dynamic constraints on an inherently active system. To demonstrate this approach, we develop a proof-of-principle model of representational differentiation, also known as structure learning, and show that structured categorical representations can emerge from constraint-based dynamics.

### 1.1. Models of Representational Differentiation

In the broader context of learning models, there are two major strands of research related to differentiation of representational categories, namely, category learning and structure learning. While these fields have an overlap in their modelling scope and methods, and are not always clearly distinguished, they also come out of different research traditions providing slightly different perspectives on what we call representational differentiation. As our primary focus here is to advance constraint-based thinking within cognitive modelling rather than directly contribute to these research traditions, we only provide a brief overview of this modelling context.

Category learning is rooted in experimental research within cognitive psychology, focusing on how people learn and judge category membership of given stimuli [11,12,13]. Computational models within this tradition, such as the SUSTAIN model [14] and the rational model of categorisation [15], primarily focus on category labelling consistent with human categorisation behaviour. However, category structure is also discussed within these frameworks, showing that the boundaries between category learning and structure learning are not always clear.

The term *structure learning* is mostly rooted in a machine learning tradition, and is typically framed as a process of selecting or inferring an underlying structure of entities or categories [16]. For structure learning in Bayesian network analysis, the goal is to infer the most probable set of dependencies among a fixed set of variables [17], whereas within the active causal learning paradigm, structure is inferred through intervention in a system of covariant variables [18]. Such approaches focus on modelling a latent structure of pre-labelled data, i.e., relations between known categories. However, since the world does not present itself with labels, the structure learning of biological cognitive systems must include the more fundamental challenge of identifying the natural categories of the world.

Although recent computational cognitive modelling of structure learning spans different cognitive domains and varies in scope, many include the discovery of novel categories [19,20,21,22,23], blurring the distinction between category learning and structure learning. In these works, the term *category* might have a slightly different conceptual framing, often interpreted as either object class, abstraction, perceptual concept, or latent cause, but is, regardless of interpretation, modelled as a functional component comprising a set of parameters, which are added to or developed within a larger structure of components during learning. The unique parametrisation of each component describes the “thing” or concept in the environment that it perceptually represents, determining the likelihood of each observation belonging to the given component. The inferred structure then comprises a differentiation of the observation space into discrete categorical representations. In this sense, structure learning is the more general term for representational differentiation in learning models, as it naturally includes the learning of categories. However, in this tradition, structure learning is often contrasted with *parameter* or *parametric* learning, which refers to the learning of the individual component parameters (e.g., see [24]).

A popular class of models for structure learning is non-parameteric Bayesian models [25], in particular, Dirichlet process mixture models (DPMMs) [26,27]. These perform inference over a space of unbounded mixture components, placing a prior over the number of components. The challenge of inferring over this infinite space is made tractable by a sequential assignment process where assignment $z_{i}$ of observation $x_{i}$ to component *k* are inferred by evaluating a posterior over component membership for each observation:

(1) $$p\left(z_{n}=k|x_{n},z_{1:n-1}\right)\propto \underset{\mathrm{likelihood}}{\underset{︸}{p(x_{n}|z_{n}=k,z_{1:n-1})}}\cdot \underset{\mathrm{structural}\;\mathrm{prior}}{\underset{︸}{p(z_{n}=k|z_{1:n-1})}}$$

where the likelihood is the posterior predictive of the component given observations already assigned to it and the structural prior encodes beliefs about how component assignments should be distributed. In DPMM, the structural prior is given by the Chinese Restaurant Process (CRP), which favours assigning observations to already populated components proportionally to their size (i.e., number of assignments) while allowing for new components to form with probability proportional to a concentration parameter α:

(2) $$p\left(z_{n}=k|z_{1:n-1}\right)\propto \left\{\begin{array}{cc} m_{k} & \mathrm{existing}\;\mathrm{component}\\ \alpha & \mathrm{new}\;\mathrm{component} \end{array}\right.$$

where $m_{k}$ is the number of observations currently assigned to component *k*. Traditionally, this has been used as a way to sample from the posterior over cluster assignments by cycling through observations repeatedly until convergence.

The sequential nature of the CRP prior makes it naturally suited for online inference, i.e., incremental learning from a stream of observations, allowing for the model to expand with the complexity of incoming observations. This property has been used to model a range of cognitive phenomena [28,29,30,31]. However, model contraction does not naturally follow from this process and requires the reassignment of past observations, making it incompatible with strictly incremental inference. While Teng et al. [31] introduce a reporting-stage process for component reduction during model fitting, contraction directly during structure learning remains a challenge.

These limitations reflect a broader characteristic of approaches that require the space of possible structures to be pre-specified, whether as a prior over component assignments or as the form of a generative model (as in active inference approaches [21]). In such approaches, learning amounts to selecting or optimising within some structural space, rather than allowing for structure to emerge through ongoing system dynamics. Thus, Bayesian approaches are typically not formulated to capture the temporally extended dynamics of structure formation and how these shape learning trajectories under environmental ambiguity, which we take to be key aspects of biological cognition. In the next sections, we outline the conceptual and theoretical background for our constraint-based approach before providing a general proof-of-principle for modelling structure learning with this approach.

### 1.2. Constraints and Possibility Spaces

Our constraint-based approach is informed by work within theoretical neuroscience [32,33,34], complex systems theory [35], theoretical biology [1,2,3,4], and philosophy [5,6,36,37] that emphasises the role of constraints in adaptive dynamical systems. Constraints come in many forms. Scaffolds, buffers, attractors, entrenchments, boundaries, initial conditions, and priors are all examples of constraints, since they limit some state, process, entity, or event. Constraints are relationships. Something is a scaffold for the process it supports, a boundary for the domain it delimits, or a prior for the inference it informs. If one respectively takes away the process, domain, or inference in these examples, one leaves no scaffold, boundary, or prior.

In more general terms, constraints can be thought of as a relationship limiting a certain possibility space, either by making some possibilities less likely or by exclusion of possibilities. One straightforward way to distinguish between types of constraints is to determine what types of possibility spaces they limit. For example, explanations across Marr’s levels of analysis [38] are often said to constrain each other, in the sense that an explanation at one level excludes certain explanations at another. Here, it is a space of possible explanations that is limited. Contrast this to a hierarchical Bayesian model, where priors at an upper level constrain the space of possible parameter values at lower levels. Despite the resemblance of inter-level constraint, these two examples depict categorically different possibility spaces and therefore categorically different types of constraints in this respect.

Although limitation *is* a defining feature of constraints, their effects go beyond. Constraints can also enable new possibilities as a consequence of being limiting. As an intuitive example, games like chess are defined by strict limitations in the space of possible moves for each piece. The possibility of the game itself and all its openings and strategies is enabled by these limitations. If we gave players the freedom to move their pieces anywhere at any time, there would be no game of chess. Similarly, traffic lights enable efficient traffic flow by selectively limiting the movement of cars, and our skeleton enables our capacity to walk by limiting the possible movement of our joints [35]. The commonality here is that by constraining elements at one level, these elements are stabilised into otherwise unlikely patterns or structures necessary for realising a phenomenon at a different level.

Constraints also account for downwards causation, in the sense of a whole causing its parts [5,39,40,41]. This is a deep philosophical topic, but for our present purposes it is sufficient to note that system-level organisation can causally influence trajectories of a system’s parts in a way that cannot be reduced to part-to-part relationships. The general principle of this is that the systems organisation itself limits the space of possible trajectories of its parts and thus plays a causal selection role by excluding alternative trajectories. This may be the principle by which lower level neural dynamics can be controlled by higher-level cognitive processes. As such, constraints play an important role for emergent capacities, and in biological systems such capacities include self-organisation, agency, and cognition.

### 1.3. Bayesian Constraint Satisfaction

In probabilistic terms, relationships can be expressed in conditional probabilities, allowing for utilising a Bayesian formalism to model dynamic constraints. However, we explicitly distinguish this from the more common notion of Bayesian *inference*. In Bayesian inference, Bayes’ theorem is understood epistemically as a formal rule for optimally integrating new evidence with prior knowledge according to the rules of probability. When modelling cognitive agents as Bayesian inference agents, this rule is often understood as a temporal sequence starting with a prior that is evaluated against some evidence using the likelihood, then yielding a posterior. This maps a certain procedure for calculating a conditional probability onto a temporal sequence of an epistemic inference procedure that the cognitive agent is performing. However, the relationships between the quantities that Bayes’ theorem consists of are not inherently temporal or sequential, but logical. Thus, the epistemic interpretation given by Bayesian inference adds a layer of assumptions about processual relationships between epistemic entities that are not given by Bayesian logic alone.

Bayesian logic can be seen as a general a framework for probabilistically expressing how constraints are satisfied. Here, constraint *satisfaction* simply refers to how a possibility space is probabilistically limited given a constraining relationship. In this sense, Bayesian inference is the special case when the constrain satisfaction is specifically *epistemic*. In contrast, we propose to use Bayesian logic to express constraint satisfaction under a *dynamic* interpretation. This conceptually reframes the elements of Bayes’ theorem as non-sequential and non-epistemic, i.e., as quantities whose relationships define the logical conditions under which a set of constraints is satisfied at a given moment in time. In this sense, these quantities (e.g., “prior” and “likelihood”) are not necessarily viewed as concrete system entities, such as an explicit belief, but may just be modelling quantities used for computational modelling procedures.

We note that this account of constraint satisfaction is different from mathematical constraint satisfaction problems [42], as well as the use of the term in the connectionist tradition [43,44] and related works (e.g., [45]). The differences become apparent when considering what possibility spaces and what type of relationships are in focus for these different uses of *constraint* (e.g., solution spaces and relationships between network nodes in the form of connection weights).

We further note that, in the context of biological cognition, Bayesian inference is sometimes viewed as explicitly calculated or approximated by some algorithm carried out by the brain (e.g., see [29,46]). This invites researchers to view the calculations performed in cognitive models as capturing something more or less concrete about how the brain carries out its activities. While the specific computational procedure introduced in this paper is essentially agnostic to this, the dynamic framing does suggest a different picture where computation is a modelling tool, but not a modelling target (not unlike how computational modelling is used more broadly in the natural sciences). However, as will become apparent below, our approach does not exclude explicit computation or inference from biological cognition, but adds a modelling layer targeting how aspects of cognition may arise from the organisation of such processes rather than constituting additional explicit computation. We emphasise this point to clarify how we attempt to align cognitive modelling with the ideas of constraints in biological organisation.

This line of thought has led us to the design of our current structure learning model, and while the resulting computational procedure does not wholly contradict a standard epistemic interpretation (i.e., as explicit inference), we provide this perspective throughout the paper to clarify the modelling assumptions, motivate the model design, and highlight how constraint-based interpretations may offer an alternative framing for modelling cognitive systems.

### 1.4. A Constraint-Based Approach to Structure Learning

Rather than treating structure learning as the selection among candidate representations, we model it as a process in which representational structure is continually shaped by constraint-based dynamics. The goal is not to develop a comprehensive cognitive architecture, but to demonstrate that internal differentiation of perceptual categories can emerge as a result of such dynamics.

Our present purpose is to model structure learning as an emergent capacity enabled by system-level constraints on lower-level learning processes. To make our claims more precise and assessable, we introduce a set of stipulative operational definitions for the present modelling context. A *dynamic constraint* is a relationship between model entities that limits the possible time-course trajectories of one or more of the involved entities. This is different from other types of modelling constraints such as parameter fixation or limited computational resources. *Emergence* refers to possibilities enabled by dynamic constraints, e.g., trajectories that would not be stably possible without persistent exclusion of alternative trajectories. An *emergent capacity* is an emergent organisation of trajectories where the organisation poses a dynamic constraint back onto the processes enabling its emergence (i.e., a whole-to-part relationship), such that this organisation keeps emerging in a specific way or at specific times. This “specific way or time” of continued emergence is then what characterises what it is a capacity of. Thus, by characterising structure learning as an emergent capacity in this context, we mean (1) that the individual learning trajectories of a set of component processes are emergent in the sense that these trajectories would not persist absent of dynamic constraints, (2) that these emergent trajectories are organised such that each component differentiate observations of an environmental category, and (3) that this organisation itself dynamically constrains the components such that this type of organisation keeps emerging.

We distinguish between *component-level* and *system-level* perspectives, both conceptually and formally. The component-level describes individual learning processes in isolation, whereas the system-level describes the ensemble of components and their organisation. It is at the system-level that we apply our constraint-based framing to model emergent organisation and its effect on the components. Each component at the component level is itself an isolated Bayesian inference model in the conventional sense.

In this work, observations play different roles at the two levels. At the component-level, observations provide evidence for learning in the typical sense of Bayesian inference. Importantly, each component is in itself inherently active, meaning that, as a baseline, they fully learn from all observations. Thus, void of constraints, the learning trajectories of multiple components are expected to converge. At the system level, observations instead function as external constraints on the activity of each component, reflected in a limitation on the extent of component-level updates. As a consequence, even though all learning processes at the component-level “see” all observations the same, they do not all learn equally from them. Learning is dynamically constrained, such that, for a given observation, some components update strongly while others update weakly or not at all, resulting in emergent possibilities for the individual learning trajectories as they diverge. Here, categorisation is an emergent system-level property, defined by differentiation within the ensemble. Because each component functions in isolation at the component-level with equal access to all observations, no individual component can by itself carve the observation space into distinct categories. This differentiation arises only at the system-level, where the meaning of a component can be defined within the organisation in opposition to other components: it is what it is not.

**Figure 1:**
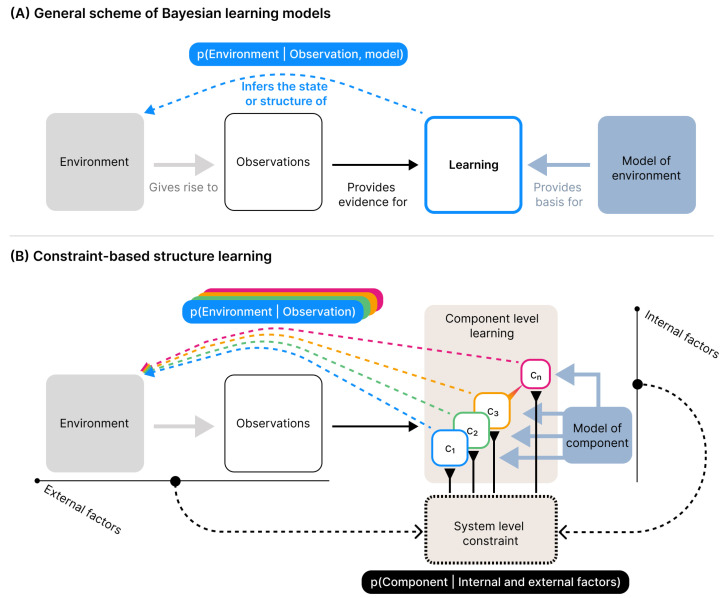
Conceptual schemes of the learning models. (**A**) The general scheme of Bayesian learning models. Here, learning is about inferring the state (or structure in the case of structure learning) of the environment. This is done using observations as evidence for the inference scheme on the basis of a model of how the environment generates these observations. Thus, the model tries to mirror the generative process of the environment, and for this reason is called a generative model. This general scheme also applies to Bayesian structure learning, where the generative model additionally includes the way structure is generated. Note that, while the inference is here expressed in simplistic form as the probability of environment-given observations, it is in principle always conditioned on the model as well. (**B**) Scheme of our constraint-based structure learning model. Here, instead of having one learning process with a generative model of the environmental structure, we have a system of component learning processes, denoted c. Each component follows the general scheme of Bayesian learning, but instead of having a generative model of the environment as a whole, it has a model of environmental components, i.e., a model of how an individual “thing” generates observations. All components receive all observations, but how much they learn from them is constrained from the system-level by both internal and external factors. Here, the constraint on the components is represented by the probability of the component given these internal and external factors. As this is not understood in terms of inference, we read it as an expression of how much the learning activity of the components are suppressed by these constraints. The structure learning unfolds dynamically, as the component learning processes are constrained into a representational structure of the environment.

Importantly, this means that the capacity we aim to model, i.e., structure learning, is not directly present in the formal description of the model, as it would be in more standard Bayesian inference models (see Figure 1). The set of equations and the algorithmic procedure we present instead specify interaction dynamics and constraints, and structure learning is solely modelled through the unfolding of these dynamics over time, i.e., by forward simulation. In line with our operational definitions above, structure learning as an emergent capacity is evaluated by the continued emergent organisation of the component learning trajectories during simulation.

The model we present here treats structure learning as the result of ongoing interactions between internal and external constraints. Internal constraints capture the limitations imposed on the system dynamics from within, thus referring to the system’s internal context, while external constraints are limitations imposed by the system’s external context. In the present implementation, the external constraint is given by the current observation, but more generally, external constraints may reflect any factor in the external context. Likewise, internal constraints may in principle be operationalised differently from how we do so here without violating the general approach. The central premise is that, through the interaction of these internal and external constraints, the system continually reshapes itself through local dynamics within its developing structure, rather than selecting among discrete representational alternatives. Here, a stable set of representational categories emerges as some representations becoming increasingly influential over time, while others fade.

This approach turns out to be particularly useful when considering structure learning in ambiguous environments that cannot be truly and fully differentiated, in principle. In the following section, we provide a formal specification of the model and describe the simulation environment, experiments, and evaluation measures used in this study.

## 2. Materials and Methods

The model consists of a set of components added incrementally over time. Each component implements an individual learning process that tracks the central tendency and variability of a stream of observations. We distinguish between a *component-level* description of the internal learning processes and a *system-level* description of the ensemble, which governs component activity and determines how components contribute to the evolving representational structure.

**Figure 2:**
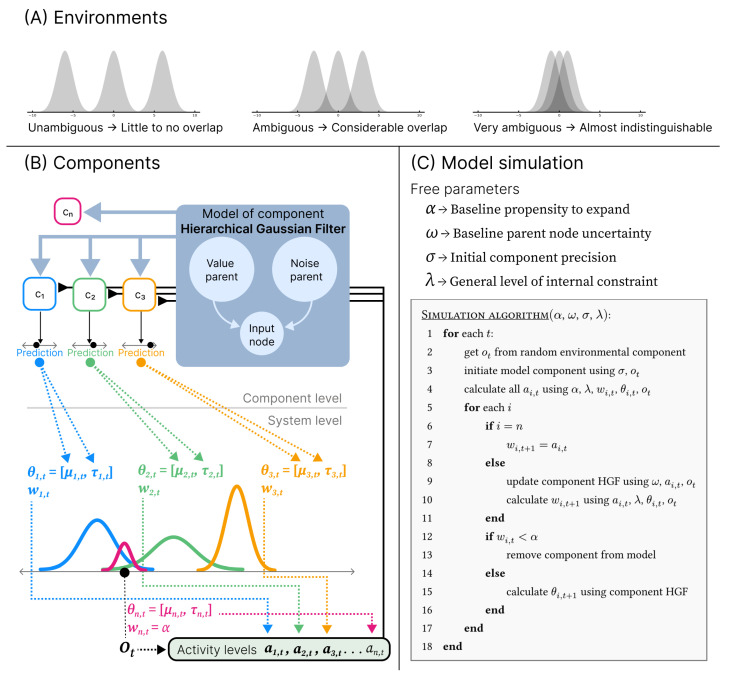
Overview of the simulation environments and model procedure. (**A**) Depiction of environments. Each Gaussian distribution correspond to a single environmental component. The depicted environments consists of three components of varying ambiguities, which are defined by the amount of overlap between environmental components. The three environments depicted correspond to the unambiguous, ambiguous, and very ambiguous environments used in the simulation experiments. (**B**) Schematic of model components and the relation between component-level learning and system-level organisation. At the component level, each component is an isolated instance of an individual learning process implemented here as a simple Hierarchical Gaussian Filter (HGF) serving as a generative model of an environmental component. Every timestep, the HGFs produces a prediction about incoming observations. At the system level, each component is represented as a candidate category with a Gaussian representational capacity distribution over the observation space, parametrised by $\theta_{i,t}=\left[\mu_{i,t},\tau_{i,t}\right]$, and a weight $w_{i,t}$, where $\theta_{i,t}$ is derived from the current HGF predictions (depicted by coloured dashed lines traversing from component-level to system-level). At each timestep *t*, a new component $c_{n}$ is instantiated with representational capacity centred on the observation $o_{t}$, representing a candidate expansion of the representational structure. A distribution of activity levels $a_{i,t}$ across components is calculated from the representational capacity of components and the observation (depicted by the arrows pointing to the activity levels box). These activity levels modulate learning by scaling the component-level update of each established HGF (depicted by the solid black lines with a reverse arrowhead; note that they continue behind the HGF box). (**C**) Free parameters and pseudocode for the simulation algorithm.

The model receives a stream of one-dimensional real-valued observations $o_{t}\in R$ over discrete timesteps *t*. Observations are generated by a set of environmental sources, each associated with a distinct distribution in the observation space. Each such distribution is referred to as an *environmental component*, i.e., a “thing” that gives rise to observations in a particular region of the observation space. At each timestep, a single observation is generated by a randomly selected environmental component, after which it becomes inaccessible. While the environment has a stable underlying structure, the model is exposed to it one observation at a time, and the generating source of any given observation is latent. Depending on the distance between environmental components, observations may therefore be more or less ambiguous due to overlaps in observation space (see Figure 2A).

At each timestep, the model encounters an observation $o_{t}$ and proceeds through the following update cycle:

1. A new component is instantiated and added to the model, anchored on the current observation.
2. At the system level, an activity level is computed for each component.
3. At the component level, established components update their learning state proportionally to their activity level (the newly added component is excluded from this step).
4. Components with insufficient weight are removed from the model.

The overall model structure is formed by repeating this cycle over time, allowing for components to be added, updated, and removed throughout learning.

The model includes three system-level free parameters and the free parameters that define the component-level. For orientation, we summarise the roles of all parameters here before specifying their precise effects below. At the system level, α sets the threshold for component removal and determines a baseline propensity for structural expansion. This propensity is further modulated by σ and λ, which respectively control the initial precision of newly instantiated components’ representational capacity and the general level of internal constraint. To model the component-level learning process, various learning models can be used. Here, we use the Hierarchical Gaussian Filter (HGF), described by one free parameter ω. Unless otherwise specified, we use the following standard parametrisation throughout the simulations:

$$\begin{array}{cc} \sigma & =0\\ \lambda & =0.7\\ \alpha & =0.01\\ \omega & =-6 \end{array}$$

These values were chosen based on exploratory tests and visual inspection aimed at identifying a region of the parameter space in which the model exhibits stable behaviour across conditions.

### 2.1. Component-Level

To describe the learning process at the component-level we use the HGF, a generalized modelling framework for inverting networks of hierarchically coupled random walks (refer to [47] for details).

Here, we use a simple HGF structure consisting of a single continuous input node with a value parent and a noise parent. The value parent tracks the central location of the observation in observation space while the noise parent tracks the variability of the observations. At each timestep *t*, the input node of the *i*-th component makes a prediction about the mean and precision of the observations at the next timestep t+1, based on the predictions of the parents:

(3) $${\hat{\mu }}_{i,t}^{\mathrm{input}}={\hat{\mu }}_{i,t}^{\mathrm{value}}$$

(4) $${\hat{\pi }}_{i,t}^{\mathrm{input}}=\frac{1}{\exp(\zeta_{i}^{\mathrm{input}}+\mu_{i,t-1}^{\mathrm{noise}})}$$

Here the superscript denotes what HGF node the variable belongs to and hat denotes that it is a prediction. Note that $\zeta_{i}^{\mathrm{input}}$ is a constant denoting the input node’s tonic observation noise, and with Equation (4) controls its baseline precision prediction. Parent predictions are computed similarly for both parent types:

(5) $${\hat{\mu }}_{i,t}^{\mathrm{parent}}=\mu_{i,t-1}^{\mathrm{parent}}$$

(6) $${\hat{\pi }}_{i,t}^{\mathrm{parent}}=\frac{1}{\frac{1}{\pi_{i,t-1}^{\mathrm{parent}}}+e^{\omega }}$$

where ω is an HGF parameter which controls the baseline uncertainty of the parent, which in turn controls the learning rate. This could in principle vary across nodes, but in our model, we set ω as a global fixed parameter for all nodes. The mean and precision of both parent nodes are updated at every timestep:

(7) $$\pi_{i,t}^{\mathrm{value}}={\hat{\pi }}_{i,t}^{\mathrm{value}}+{\hat{\pi }}_{i,t}^{\mathrm{input}}a_{i,t}$$

(8) $$\mu_{i,t}^{\mathrm{value}}={\hat{\mu }}_{i,t}^{\mathrm{value}}+\frac{{\hat{\pi }}_{i,t}^{\mathrm{input}}}{\pi_{i,t}^{\mathrm{value}}}\delta_{i,t}a_{i,t}$$

(9) $$\pi_{i,t}^{\mathrm{noise}}={\hat{\pi }}_{i,t}^{\mathrm{noise}}+\frac{1+\epsilon_{i,t}}{2}a_{i,t}$$

(10) $$\mu_{i,t}^{\mathrm{noise}}={\hat{\mu }}_{i,t}^{\mathrm{noise}}+\frac{\epsilon_{i,t}}{2\pi_{i,t}^{\mathrm{noise}}}a_{i,t}$$

where δ and ϵ denotes value and noise prediction error, respectively:

(11) $$\delta_{i,t}=o_{t}-{\hat{\mu }}_{i,t}^{\mathrm{input}}$$

(12) $$\epsilon_{i,t}=\frac{{\hat{\pi }}_{i,t}^{\mathrm{input}}}{\pi_{i,t}^{\mathrm{value}}}+{\hat{\pi }}_{i,t}^{\mathrm{input}}\delta_{i,t}^{2}-1$$

The variable $a_{i,t}$ denotes the activity level for the *i*th component at time *t* calculated at the system-level. This variable is not part of the HGF framework, but an addition to the update equations, which is central to our current modelling approach. Note that the form of some of the above equations, as well as the updating narrative, has been simplified in the light of our modelling context. For a full account, refer to [47]. However, besides adding the activity term $a_{i,t}$ the above is mathematically equivalent with the HGF framework.

New components are initialised with the following values:

(13) $$\begin{array}{cc} \zeta_{n}^{\mathrm{input}} & =\sigma \end{array}$$

(14) $$\begin{array}{cc} \mu_{n,t}^{\mathrm{value}} & =o_{t} \end{array}$$

(15) $$\begin{array}{cc} \pi_{n,t}^{\mathrm{value}} & =1 \end{array}$$

(16) $$\begin{array}{cc} \mu_{n,t}^{\mathrm{noise}} & =0 \end{array}$$

(17) $$\begin{array}{cc} \pi_{n,t}^{\mathrm{noise}} & =1 \end{array}$$

where *n* denotes the number of components and consequently, as a subscript, also denotes the newest added component. Here, σ is a global system-level parameter. Note that in the case that σ changes during the course of learning, it is possible for different components to have different values of $\zeta_{i}^{\mathrm{input}}$, as they inherit the σ value at the time of their initialisation. However, while $\zeta_{i}^{\mathrm{input}}$ is constant, it is always used in summation with $\mu_{i}^{\mathrm{noise}}$ (Equation (4)), and can therefore be seen as setting an initial condition, i.e., a constraint that the learning process can overcome by adjusting $\mu_{i}^{\mathrm{noise}}$ relative to $\zeta_{i}^{\mathrm{input}}$. Or in epistemic terms, σ functions as a prior precision for new components at the component-level, specified at the system-level.

### 2.2. System-Level

In the previous section, we describe the individual learning processes at the component-level. Now we turn to the system-level and start by describing how components are viewed at this level. We note that, at this level, it is irrelevant for the general form of the model what learning processes are implemented at the component-level, although some specifics are tailored to work with the HGF in this instance of the model (e.g., σ).

#### 2.2.1. Components

Each model component functions as a candidate representational category, comprising a Gaussian distribution over the observation space. This distribution characterises the *representational capacity* of the component, i.e., the degree to which a component is able to represent an observation at a given location in the observation space. The *i*th component’s representational capacity at time *t* consists of a Gaussian distribution parametrised by $\theta_{i,t}=\left[\mu_{i,t},\tau_{i,t}\right]$ and a weight $w_{i,t}$. Here, $\theta_{i,t}$ is derived from the learning process at the component-level, or in this instance, the predictions of the input node:

(18) $$\begin{array}{cc} \mu_{i,t} & ={\hat{\mu }}_{i,t}^{\mathrm{input}} \end{array}$$

(19) $$\begin{array}{cc} \tau_{i,t} & ={\hat{\pi }}_{i,t}^{\mathrm{input}} \end{array}$$

We use τ to represent the precision of the representational capacity distribution and π to represent precision within the HGF framework to emphasise the conceptual difference. Where π is understood as an estimate of uncertainty, τ is related to the component’s system-level representational scope in observation space. Thus, low values of τ are not interpreted as the model being uncertain about the representational category. Since $\tau_{i,t}$ is directly derived from the precision prediction of the input node, whose initial condition is in effect set by σ (as described above), at the system-level, we can understand σ as controlling the initial value of $\tau_{n,t}$.

The weight $w_{i,t}$ is a quantity representing the relative magnitude of the *i*th component’s representational capacity on the system-level. This means that the model can differentially weight components irrespective of their representational scope in observation space (given by θ), thereby allowing for differentiation in the representational space of components that do not differ in terms of θ, i.e., that cover the same area of observation space. As we shall see, this is crucial to the adaptability of the structure learning dynamic.

We use the notation $c_{i}$ to refer to the *i*th component as a whole. At the system-level, $c_{i,t}$ is the representational capacity given by the combination of $\theta_{i,t}$ and $w_{i,t}$.

#### 2.2.2. Activity Level and Weight Update

At each timestep *t*, the model is exposed to a new observation $o_{t}$, which constrains the update of the components. Before updating each component, an activity level $a_{i,t}\in \left[0,1\right]$ is computed. The full set of activity levels at time *t* forms a categorical probability distribution over components:

(20) $$\sum_{i}a_{i,t}=1$$

We define each probability $a_{i,t}$ as the probability of the *i*th component given a set of internal and external constraining factors, which can be expressed in the general form:

(21) $$a_{i}=p\left(c_{i}|I,E\right)$$

where *I* represents some internal constraint posed from within the system and *E* represents an external constraint posed by the environment. As such $a_{i}$ represents the simultaneous satisfaction of those constraints on the learning activity of the component, where a value of 1 means no limitation and a value of 0 means full exclusion of the activity. At a specific timestep, we here keep the internal constraint arbitrarily defined (this is further discussed below) while taking the observation to be the externally constraining factor:

(22) $$E_{t}=o_{t}$$

In the following, we use $o_{t}$ to denote the external constraint for clarity in relation to our current implementation, where only *sensory* constraints are posed externally. However, theoretically, *E* could represent any number of external factors, e.g., *physical* or *social* constraints.

To calculate $a_{i,t}$, we use Bayes’ theorem to decompose the conditional probability into the following proportionality:

(23) $$p\left(c_{i,t}|I,o_{t}\right)\propto p\left(I|c_{i,t},o_{t}\right)\cdot p\left(c_{i,t}|o_{t}\right)\propto p\left(I|c_{i,t},o_{t}\right)\cdot p\left(o_{t}|c_{i,t}\right)\cdot p\left(c_{i,t}\right)$$

We define the marginal probability of the component in terms of component weight:

(24) $$p\left(c_{i,t}\right)=\frac{w_{i,t}}{\sum_{j}w_{j,t}}$$

We define the probability of the component given the observation with the probability density function for the Gaussian distribution *f*:

(25) $$p\left(o_{t}|c_{i,t}\right)=\frac{f(o_{t},\theta_{i,t})}{\sum_{j}f\left(o_{t},\theta_{j,t}\right)}$$

This leaves us to define the term $p(I|c_{i,t},o_{t})$. As *I* can be arbitrarily defined, we conceptualise this term as a probabilistic expression of a system-wide constraint affecting each component individually, which is in turn locally constrained by the interaction between the given component and the external constraint (here the observation). We formally define it as given by the function *g*:

(26) $$p\left(I|c_{i,t},o_{t}\right)=g\left(\lambda ,o_{t},\theta_{i,t}\right)=\frac{2}{\pi }\arctan\left(\frac{e^{\lambda }}{{\left(o_{t}-\mu_{i,t}\right)}^{2}\tau_{i,t}}\right)$$

where λ is a global model parameter that inversely scales the general level of internal constraint *I* across all components, i.e., when λ is large, components are generally less constrained. The term ${\left(o_{t}-\mu_{i,t}\right)}^{2}\tau_{i,t}$ is constructed to express the interaction between the component and the external constraint on which *I* is conditioned. This is a modelling choice that ensures that internal constraint varies dynamically with the component-level learning process, such that it remains component-specific rather than being rescaled relative to the full ensemble. The ratio $e^{\lambda }/\left({\left(o_{t}-\mu_{i,t}\right)}^{2}\tau_{i,t}\right)$ is translated into probability space using $\frac{2}{\pi }\arctan$ (see Appendix B for an analysis of alternative mapping functions). Here, we note that π represents the common constant π≈3.14 and should be dissociated from the use of the same letter in the HGF equations. Taken together, the function *g* is a probabilistic expression that decreases the further away the observation is from the component’s concentration of representational capacity in the observation space, where λ functions analogously to a temperature parameter. The functional point of this term is that it is a structure independent (i.e., independent of other components) scaling of the relevance of the observation. This is vital for the dynamic, as it enables the constraining of components for which the observation is not relevant, and this is the basic mechanism of representational differentiation in the model. In this sense, λ can be understood as controlling the diffusion of potential relevance in the observation space relative to the representational capacity of a component.

Finally, we can express the activity level for the *i*th component at time *t* as

(27) $$a_{i,t}=\frac{g\left(\lambda ,o_{t},\theta_{i,t}\right)f\left(o_{t},\theta_{i,t}\right)w_{i,t}}{\sum_{j}g\left(\lambda ,o_{t},\theta_{j,t}\right)f\left(o_{t},\theta_{j,t}\right)w_{j,t}}$$

At every timestep, the weight of each component is updated towards the current activity level scaled by the function *g*:

(28) $$w_{i,t+1}=w_{i,t}+g\left(\lambda ,o_{t},\theta_{i,t}\right)\left(a_{i,t}-w_{i,t}\right)$$

In this way, the weight represents a trace of the component’s activity history dynamically modulated by the internal constraint and the component’s own interaction with the environment. This effectively means that the weight reflects the activity history in proportion to the representational capacity of the component throughout that history.

In summary, $a_{i}$ represents how external and internal constraints on component activity are jointly satisfied. This constraint satisfaction is calculated from the following three quantities: $g(\lambda ,o_{t},\theta_{i,t})$, $f(o_{t},\theta_{i,t})$, and $w_{i,t}$. As noted in the Introduction, these quantities are not necessarily seen as system entities, here meaning that they are just the computable logical decomposition of the total constraint satisfaction $a_{i}$. The exception is $w_{i,t}$, which is understood as the relative magnitude of the component representational capacity. We note that the specific form of $g(\lambda ,o_{t},\theta_{i,t})$, $f(o_{t},\theta_{i,t})$, and the update equation for $w_{i,t+1}$ are non-arbitrary modelling choices. While system-level and organisational constraint could potentially be operationalised and modelled in many ways, we have constructed the above from a Bayesian perspective on constraint satisfaction and designed it to represent organisational tensions as a model of the emergence of structure learning (see also below). Here, an important aspect is that the weights can update in both positive and negative directions, enabling dynamic model contraction in addition to expansion. Next, we describe these aspects of the model.

#### 2.2.3. Adding and Removing Components

Recall that the model’s structure evolves continuously as the result of an iterative update cycle, where all components learn in parallel at the component-level. At each timestep, the update cycle includes the following stages in sequence: (1) A new component is added. (2) Activity is calculated for all components. (3) HGFs and weights are updated for all but the new component. (4) Components with insufficient weight are removed. At the system-level, since the constraint relations that we model are understood as momentary, the full update cycle is viewed as a model of a single event. Thus, a component that did not survive its initial update cycle can be understood as an unrealised potential. It could have come into existence during the course of the event, but it did not. As such, the form of the new component, as computationally initialised, reflects the model’s momentary propensity for model expansion, where the form it takes at the end of the update cycle represents the potentially realised component. Thus, when we refer to a *new* component, we refer to the computational entity playing the role of newly added component within the computational procedure, which are understood as something not realised in the system we model.

New components are initialised with the following weight:

(29) $$w_{n,t}=\alpha$$

Here, α∈[0,1] represents a global parameter controlling the baseline propensity for structural expansion.

The activity level and weight for a newly added component are computed differently. Per definition, when $\mu_{n,t}^{\mathrm{value}}=o_{t}$ (as it is at initialisation), then necessarily $o_{t}-\mu_{i,t}=0$, which leaves the function *g* in Equation (26) undefined due to division by zero. While it might seem sensible to define this function to be 1 for new components, as $g(\lambda ,o_{t},\theta_{i,t})$ approaches 1 when ${\left(o_{t}-\mu_{i,t}\right)}^{2}$ approaches 0, there is a conceptual gap between viewing components at this stage as yet *unrealised* and viewing this term as incorporating a *realised* interaction between the component and the environment. For this reason, and in line with interpretation of α (see below), we define $p(I|c_{n,t},o_{t})$ for new components as

(30) $$p(I|c_{n,t},o_{t})=\alpha$$

Otherwise, new components take part in the calculation of activity levels as if they were an established component. Given Equations (29) and (30), we can write $a_{n,t}$ as the following:

(31) $$a_{n,t}=p\left(o_{t}|c_{n,t}\right)\frac{\alpha^{2}}{\Sigma +\alpha^{2}}$$

where Σ is the normalisation excluding the new component:

(32) $$\Sigma =\sum_{i=1}^{n-1}g\left(\lambda ,o_{t},\theta_{i,t}\right)w_{i,t}$$

For the new component, there is no realised learning process at the component-level affected by the activity level, which is why the update step for these are skipped for new components. Instead, the weight for the next time step, i.e., the weight of the component if realised, is directly set to its activity level:

(33) $$w_{n,t+1}=a_{n,t}$$

Components with $w_{i,t+1}<\alpha$ at the end of the update cycle are removed permanently from the model, meaning that new components are removed when Σ≥α. Thus, the joint role of alpha as initial component values and removal threshold provides a baseline propensity for structural expansion further modulated by $p(o_{t}|c_{n,t})$. This ensures that only new components that gain sufficient weight during their initial update cycle are effectively added to the model, marking its realisation. This prevents the model from continuously accumulating components, while retaining the ability to dynamically expand in direct response to the environment. The larger α is, the more likely the new component is to be realised, relative to the representational capacity of other components at the same location in observation space. In this sense, α represents a general pressure to expand the model structure, which is constrained by the representational capacities already captured by the model.

We note that while we here use α as a global removal threshold for all components, the conceptual justification for interpreting it as the baseline propensity for structural expansion is only related to the removal threshold for *new* components. However, in relation to the removal of old components, α plays a different conceptual role. This means that the removal threshold of new and old components should be conceptually distinct, even if we do not make this distinction formally here.

Adding and removing components allows for both structural expansion and contraction. However, it is through the unfolding of the temporally extended dynamics that this becomes structure *learning*. Here, the notion of *tension* between components plays an important explanatory role. We briefly outline some of the dynamics related to structural expansion and contraction.

### 2.3. Tension and Contraction

The discrepancy between $g(\lambda ,o_{t},\theta_{i,t})$ (Equation (26)) and the activity level (Equation (27)) is an especially important relation driving the flexible development of the model. Since one is structurally dependent and the other structurally independent, a discrepancy such that the value of *g* is large and the activity level is small means that there are other components with representational capacity at the same location. This creates a tension between components. As all involved components will update their weights towards their respective activity level, relatively unconstrained by *g* (due to its value being large), if observations recur at roughly the same location over time, components with lower weights will lose weight faster than others and will eventually either perish or be attracted to another location in observation space. This either resolves the tension with a remaining component dominating the given location (regaining weight on subsequent timesteps) or stabilises the tension between the internal and external constraints, allowing for shared representational capacity in the given area of the observation space.

The realisation of this dynamic depends on λ, as this parameter effectively controls *g*’s (Equation (26)) sensitivity to representational capacity. For extremely large values of λ, the value of *g* will always be near 1, and, conversely, for an extremely low λ value, the value of *g* will always be near 0. This means that the effect of λ falls on a continuum, where at one end (large values), *all* component weights update maximally every timestep, i.e., becoming practically equal to the current activity level, and as λ decreases, weight updating is more and more suppressed. For the structure learning dynamic, this means that λ scales the limits of sustained tension between components, and consequently controls how much shared representational capacity or representational ambiguity is tolerated.

### 2.4. Parametrisation and Expansion

Since the representational capacity of new components is centred on the observation, the more precise the distribution is (i.e., larger $\tau_{n}$), the larger $p(o_{t}|c_{n,t})$ (Equation (25)) is in the calculation of the activity level (Equation (31)). This means that σ effectively scales the activity level of new components, such that smaller σ values amplify the propensity for structural expansion. Consequently, low σ values may result in elevated structural expansion of overly precise components, which due to their high concentration of representational capacity result in less tension between components, making it less likely that the dynamic will resolve into a proper representational structure as described above.

Low values of λ also interfere with the propensity for structural expansion, but, in a sense, for the opposite reason of that described for σ. Recall that low values of λ suppress weight updating. This is because the function *g* returns lower values when λ is decreased, but since *g* is not involved in the calculations of $p(I|c_{n,t},o_{t})$ for new components, λ has no suppressing effect here. Yet λ still plays a suppressing role for Σ in Equation (31). Consequently, low λ values elevate the propensity for structural expansion by suppressing everything but the new component, resulting in greater activity levels and thus a greater initial weight for new components.

### 2.5. The Model’s Relation to Bayesian Inference

The model formally resembles the CRP-based inference discussed in the Introduction, and it is worth making this relationship explicit. We first restate the Dirichlet Process Mixture Model (DPMM) approach in relevant detail (for a full account see [26,27]), but using notation consistent with our model equations above to allow for direct comparison.

In DPMM inference, the posterior probability of assigning observation $o_{t}$ to component *i* can be written as

(34) $$p\left(z_{t}=i|z_{1:t-1},o_{t}\right)\propto p\left(o_{t}|z_{t}=i\right)\cdot \left\{\begin{array}{cc} m_{i} & \mathrm{established}\;\mathrm{component}\\ \alpha & \mathrm{new}\;\mathrm{component} \end{array}\right.$$

where *z* is the vector of label assignments and $m_{i}$ is the number of observations currently assigned to component *i*. The activity level of the present model $a_{i}=p\left(c_{i}|I,o_{t}\right)$, given by Equations (27) and (31), can be written in a parallel form:

(35) $$p\left(c_{i}|I,o_{t}\right)\propto p\left(o_{t}|c_{i,t}\right)\cdot \left\{\begin{array}{cc} p\left(I|c_{i,t},o_{t}\right)\cdot w_{i,t} & \mathrm{established}\;\mathrm{component}\\ \alpha^{2} & \mathrm{new}\;\mathrm{component} \end{array}\right.$$

where the $\alpha^{2}$ for new components follows from α replacing both $p(I|c_{n,t},o_{t})$ and $w_{n,t}$ at initialisation (Equations (29) and (30)).

Since $p(I|c_{i,t},o_{t})$ is observation-dependent, it belongs to the likelihood rather than the prior-like term. Treating it as such gives a factored likelihood:

(36) $$p\left(I,o_{t}|c_{i,t}\right)=p\left(I|c_{i,t},o_{t}\right)\cdot p\left(o_{t}|c_{i,t}\right)$$

Now the activity can be rewritten as

(37) $$p\left(c_{i}|I,o_{t}\right)\propto p\left(I,o_{t}|c_{i,t}\right)\cdot \left\{\begin{array}{cc} w_{i,t} & \mathrm{established}\;\mathrm{component}\\ \alpha & \mathrm{new}\;\mathrm{component} \end{array}\right.$$

This is structurally identical to the CRP decomposition, with the likelihood $p(I,o_{t}|c_{i,t})$ in place of $p(o_{t}|z_{t}=i)$ and $w_{i,t}$ in place of $m_{i}$. However, $w_{i,t}$ is a continuous quantity, rather than a count variable, and the likelihood is richer than in standard DPMM, incorporating the additional term $p(I|c_{i,t},o_{t})$ defined in Equation (26).

The weight update in Equation (28) has no direct parallel in DPMM. Rather than incrementing a count upon assignment, $w_{i,t}$ is continuously updated towards the current activity level, scaled by $p(I|c_{i,t},o_{t})$. This means that weight reflects a dynamically modulated trace of activity history rather than accumulated evidence for assignment, and it is this continuous update dynamic that enables model contraction alongside expansion.

While one could treat Equation (36) as a richer likelihood term encoding both the fit and relevance of the observation, sustaining a full inference interpretation requires treating *I* as an observed quantity in the same sense as $o_{t}$. In the DPMM inference the posterior is conditioned on the assignment of past observations $z_{1:t-1}$ in addition to the current observation, which may appear similar to how $a_{i}=p\left(c_{i}|I,o_{t}\right)$ is additionally conditioned on *I*. However, $z_{1:t-1}$ is absorbed by the CRP prior and does therefore not appear in the likelihood, but due to the structure of *g* (Equation (26)) in the present model, there is no way to fully absorb *I* into a prior. Consequently, the inference framing dictates that *I* must be an observable, leaving a conceptual gap open for what *I* actually represents within an inference interpretation, which makes the translation into explicit inference less straightforward.

Furthermore, the nature of the posterior is different between the models. In DPMM inference, the posterior $p(z_{t}=i|z_{1:t-1},o_{t})$ is used to assign each observation to a component, and structural change is driven by the accumulation of these assignments. In the present model, $a_{i}=p\left(c_{i}|I,o_{t}\right)$ is used for modulating component-level learning without exclusively attributing observations to a single component.

### 2.6. Simulation Experiments and Measures

We ran a series of simulation experiments to test our model. In these experiments, we investigated various aspects of the model in terms of environmental alignment, primarily from a behavioural perspective. Such a behavioural perspective, i.e., forcing the model to classify what component represents a given observation, provides a simple way to measure structural alignment.

In all simulations, the environment consists of three environmental components of standard deviation 1, with equal distance to neighbouring components. The difference between environments, i.e., different levels of ambiguity, is given by the distance between neighbouring means. When this distance is 6, we call it *unambiguous*, as there is virtually no overlap. When the distance is 3, there is a considerable overlap, and we call it *ambiguous*. When the distance is 1, we call it *very ambiguous* (see Figure 2A for a graphical representation).

To measure the alignment between the structure of the environment and the structure learned by a model from a behavioural perspective, we used the adjusted Rand index (ARI) [48] between a model’s judgement of a set of observations (i.e., judgment about what “thing” generated them) and the environmental ground truth. The ARI is a measure developed for testing the similarity of two ways of clustering the same dataset, which is agnostic to the number of clusters in each. For the ARI, a value of 0 indicates that the similarity is at chance level, and a value of 1 indicates that they are identical. To get behavioural data from the model, we draw a sample from a categorical distribution parametrised by the distribution of activity levels calculated for a given observation. This sample serves as the model’s judgment about what “thing” generated the observation, and, for a set of observations, such judgments correspond to a clustering of the observations. When calculating the ARI, we kept the model fixed (i.e., preventing updating) and test the given state of the model for a set of observations. As such, we used a training and test phase approach. In this way, the ARI serves as a measure of a model’s potential behavioural performance for a given state.

In the training phase, the model simulation was run for a sequence of timesteps, as described in the model description. In the test phase, 20 random observations were drawn from each environmental component. For each of these observations, we get the model’s judgment as described above. Then, for each simulation, the ARI between the set of model judgements and the ground truth was calculated as a measure of the models potential behavioural performance.

To supplement the ARI measure, we provide two additional metrics: the number of components and the MW_2_ distance [49] between model and ground truth. The MW_2_ is a Wasserstein-type distance (also known as earth mover’s distance) developed specifically to measure the distance between Gaussian mixture models as an optimal transport problem. While the number of components measures a direct structural property giving a direct insight into model expansion and contraction, the MW_2_ measures the difference in combined mixture density and is not itself a measure of a structural property. Unlike the ARI, which maps onto a behavioural response, the MW_2_ has no direct psychological or behavioural interpretation and is included purely as a model analysis tool. However, it provides information about the overall density coverage of the learned components compared to the environment, illuminating an aspect of the internal model dynamics not directly covered by the structural measures or accessible from behaviour alone.

Our focus is to evaluate the models learned structure in the sense of how components are organised in relation to each other, and the measures are chosen to reflect this. The model simultaneously performs parameter learning at the component-level, i.e., learning the Gaussian means and precisions, as part of the structure learning dynamic. However, measuring this in a coherent way across conditions is not a straightforward matter, as the number of components, their parameters, and their weights varied in ways that made consistent matching difficult. Since this is not our focus, we only evaluated this aspect through visual inspection of the various examples given.

## 3. Results

**Figure 3:**
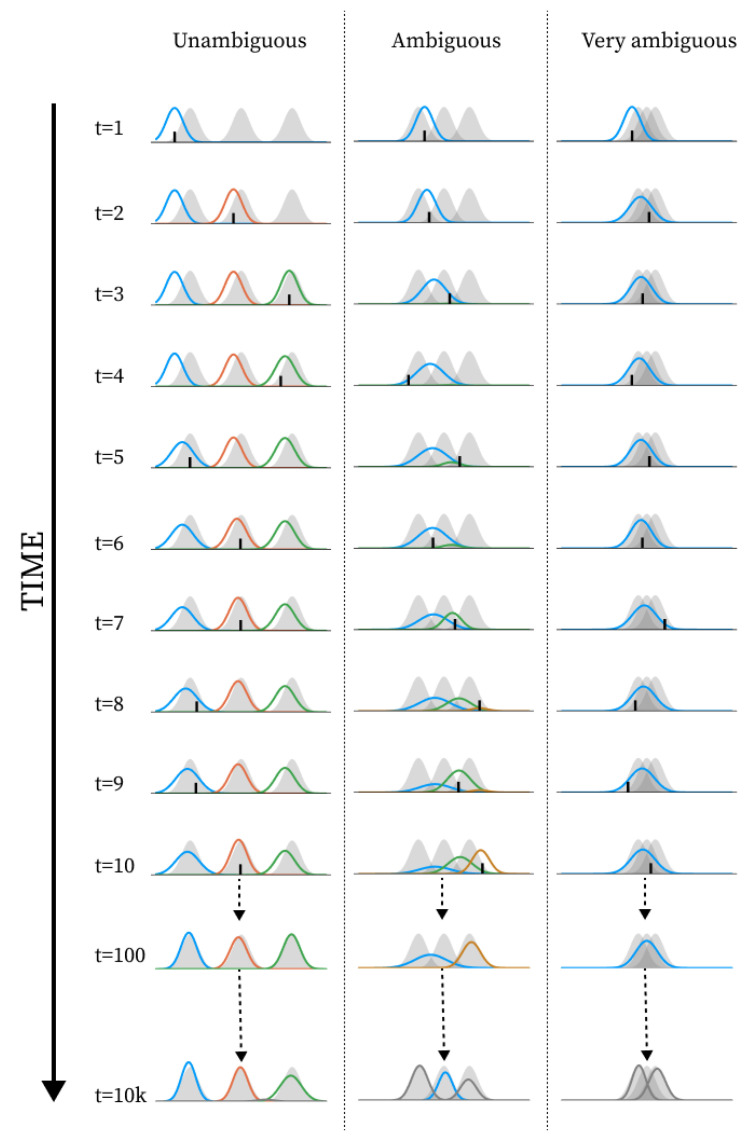
Examples of how the learning dynamic unfolds with standard parametrisation at different levels of ambiguity. The three columns of plots each represent a simulation spanning 10,000 timesteps. Every row represents a timestep denoted to the right of the row. For the first ten rows, we show the initial learning during the first ten timesteps. The last two rows represents the state of the model after 100 timesteps and at the end of the simulation. The x-axis of every plot represents the observation space. Every plot shows the environmental components as grey shaded Gaussian distributions. Each coloured line represents the distribution of representational capacity for a model component, where the height is scaled by the weight of the component. Each plot shows the state of the model after updating on the given timestep, and for the first ten timesteps, small black lines represent the observation that these updates were based on. The colour of the lines represent the timestep the component was added in within the first ten timesteps. Components added after the first ten timesteps are shown in grey. The leftmost column shows a simulation in an unambiguous environment (distance between means = 6), the middle column in an ambiguous environment (distance between means = 3), and the rightmost column in a very ambiguous environment (distance between means = 1). The examples are chosen to show typical simulation runs.

**Figure 4:**
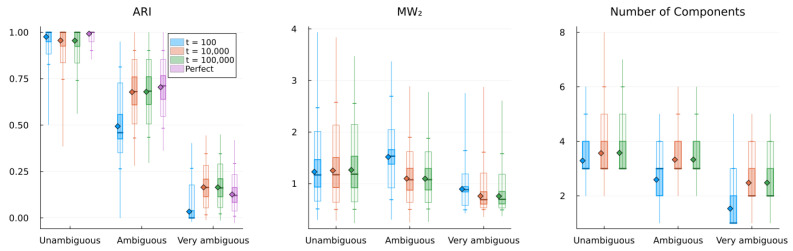
Box plots depicting the distributions of measured values from simulations of various durations and ambiguities using standard parametrisation. Each distribution is obtained from 10,000 simulation runs, and all measures are obtained at the end of simulation. The three plots represent the three measures: ARI, MW_2_, and number of components. Each box within each plot shows the following quantiles: 50% (median line), 25% and 75% (inner box), 5% and 95% (outer box), 1% and 99% (whiskers), minimum and maximum (vertical line). The diamond shapes depict the numerical mean. Boxes are grouped by levels of ambiguity: unambiguous (distance between means = 6), ambiguous (distance between means = 3), and very ambiguous (distance between means = 1). The colours represent the simulation duration. Blue represents short-term simulation (100 timesteps), red represents long-term simulation (10 k timesteps), and green represents a duration ten times longer than the long-term (100 k timesteps). The purple boxes in the ARI plot represent the ARI measure obtained with models that perfectly mirror the environment.

In this section, we show a series of simulation results produced by running the structure learning algorithm for different models (i.e., different parametrisations) in different environments. First, in Figure 3, we show examples of how the structure learning plays out at the system-level in different environments using the standard parametrisation given above. For a more comprehensive impression, we strongly encourage the reader to find animations of such examples in the Supplementary Material or to try out the interactive simulation tool built to support the communication of the model dynamics by visiting https://ilabcode.github.io/constraint-based-structure-learning/ (accessed on 27 January 2026). Second, in Figure 4, we show the distributions of values obtained from the three measures at standard parametrisation, serving as a baseline for comparison. Note that the measures have different optimal values. For ARI, 1 is optimal, for MW_2_, 0 is optimal, and for the number of components, 3 is optimal (given that we use three environmental components throughout). We then investigate the effects of σ at various levels before doing the same for λ, and finally we investigate the interaction between the two.

### 3.1. Investigating σ

**Figure 5:**
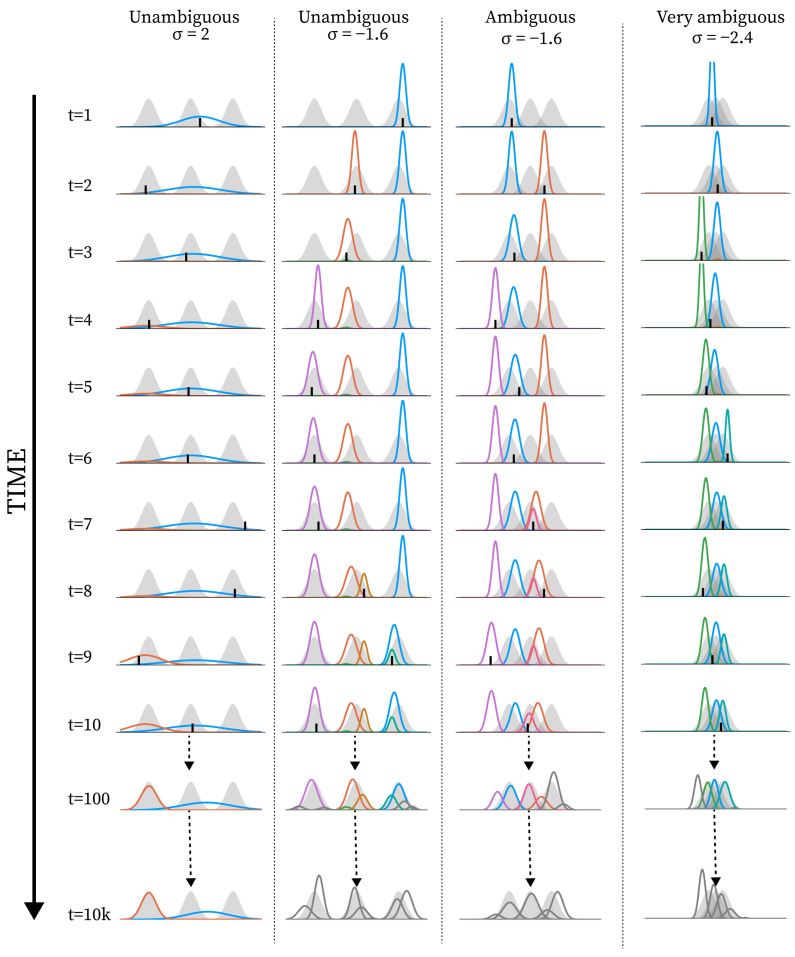
Examples of how the learning dynamic unfolds with different levels of σ at different levels of ambiguity. The plot is constructed in the same way as described in Figure 3, except that there are four columns, with the level of ambiguity and the level of σ used denoted at the top of each column.

**Figure 6:**
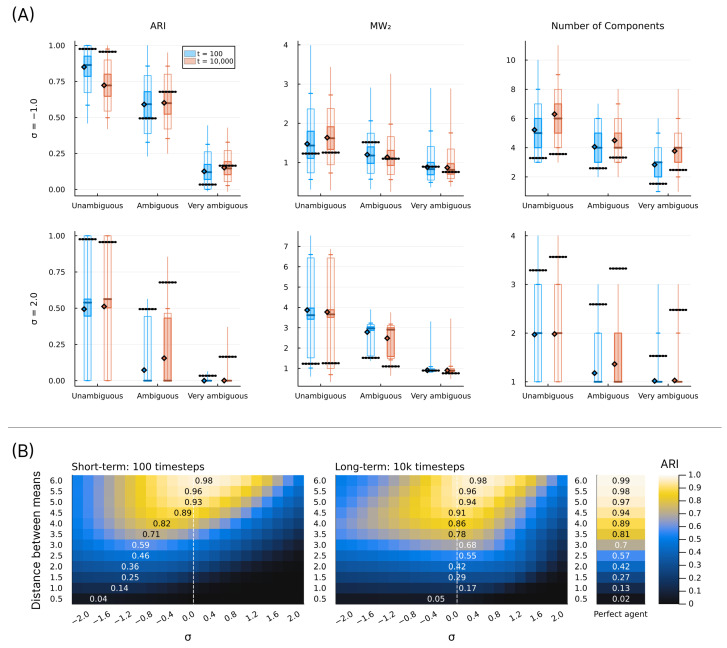
Measures at different values of σ and at different levels of ambiguity. (**A**) Box plots depicting distributions across measures. The boxes are constructed the same way as in Figure 4, with the addition of a horizontal dotted line representing the corresponding mean value at standard parametrisation (the means shown in Figure 4). The top row represents simulations using σ=−1 and the bottom row represents simulations using σ=2. (**B**) Heatmaps showing model ARI performance under varying σ values and levels of ambiguity. Each cell depicts the mean behavioural performance across 10,000 simulations using a σ value represented by the x-axis and a distance between neighbouring means given by the value on the y-axis. The mean behavioural performance is represented by a colour given by the colour gradient shown in the rightmost legend. Each simulation runs for a number of timesteps in a training phase, before behavioural performance is calculated in a test phase. One heatmap shows short-term results (a training phase of 100 timesteps) and the other shows long-term results (a training phase of 10,000 timesteps), as denoted above the heatmaps. The numbered cells represent the maximum performance value of the given row. The dashed line signifies the σ level that corresponds to new model components matching the precision of the environmental components. The column to the right depicts the results of the same simulation procedure, but using a model with components perfectly mirroring the environmental components. Note that a perfect agent is not perfect in the sense of perfect judgement, as this is in principle impossible in ambiguous environments.

In Figure 5, we see examples of how the structure learning dynamic might unfold at various values of σ diverging from the standard parametrisation. In Figure 6A, we show examples of how the measures are distributed across simulations for large and small values of σ at different levels of ambiguity. For ARI, we see a general decrease in performance compared to standard parametrisation, except for short-term performance in ambiguous environments with a decreased σ value. The MW_2_ distributions also reflect this pattern to some degree. As expected, the decreased σ value results in a generally larger number of components with the reverse pattern for the increased σ value.

In Figure 6B, we show the behavioural performance (ARI) across a range of σ values and at different levels of environmental ambiguity. Here, we see that the optimal value of σ roughly corresponds to the value matching the precision of the environmental components. However, for short-term learning, as the environment becomes more ambiguous, the optimal σ value shifts downwards, indicating that higher initial precision is preferred in ambiguous environments. In the case of long-term learning, these optima remain close to the σ value matching the environment, and here they are notably closer to the performance of perfect agents, i.e., agents with components identical to the environment.

**Figure 7:**
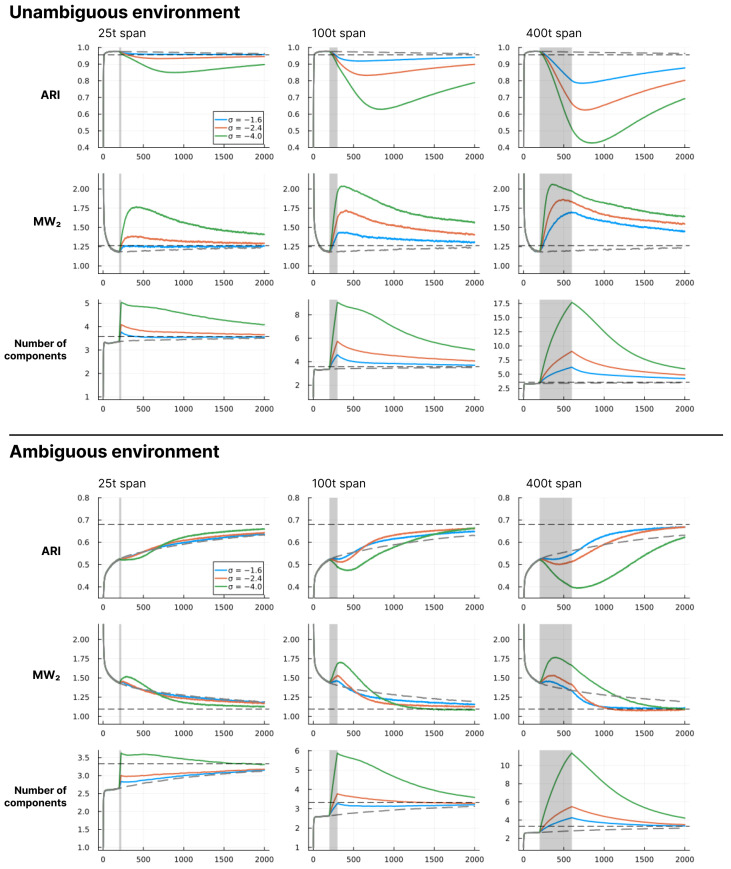
Plots showing the average trajectory of ARI, MW_2_ and number of components across two levels of ambiguity and three spans of altered σ. In each plot, for each of four tests (lines), 10,000 simulations are run for 2000 timesteps. At each timestep, each measure is obtained after the update cycle. Each line represents an average of the given measure (y-axis) across all simulations at each timestep (x-axis). The grey lines (dashed after t=200) represent tests using the standard parametrisation throughout. Other lines represent tests where parametrisation is non-standard within a time span. The grey box represents the time span (starting at t=200) during which the parametrisation diverges from the standard. Each column of plots represents a span denoted at the top of the column (spans of 25, 100, and 400 timesteps). For blue lines, σ=−1.6 within the span; for red lines, σ=−2.4; and for green lines, σ=−4. For all simulations, σ=0 before *and* after the span. The black dashed line represents the convergence point under standard parametrisation, i.e., the average performance after 100,000 timesteps. The nine plots at the top represent tests in unambiguous environments (distance between means = 6), and at the bottom, plots represent tests in ambiguous environments (distance between means = 3). Each row represents a measure denoted on the left.

In Figure 7, we see that in unambiguous environments the models reach good average performance quickly and then slowly develop towards a slightly different (worse) convergence point. Here, we see that decreasing σ within a time span has a negative effect on all measures, and the magnitude of this effect scales with both the level of σ and the length of the span. We also see that the duration of these effects extends beyond the span. For ARI, the trajectories follow a continued decrease after the span followed by a slow shift towards an increasing trend. For MW_2_, a similar pattern is observed, except the shift happens faster, and for the longest span, the shift happens already within the span. The number of components immediately starts decreasing after the span in all cases, meaning that the measured effects are not temporally aligned. For ambiguous environments, we see that models have a much slower increase towards the convergence point under standard parametrisation. Here, we see a similar pattern of negative effects for decreasing σ, but only within the time span. After the span, the effect is an overall increase in how fast the models approach the convergence point for ARI and MW_2_ on average, with some combinations of σ values and span length being better than others. Here, it seems that it is the combinations that result in an average number of components just above the convergence point at the end of the span that are preferable.

### 3.2. Investigating λ

**Figure 8:**
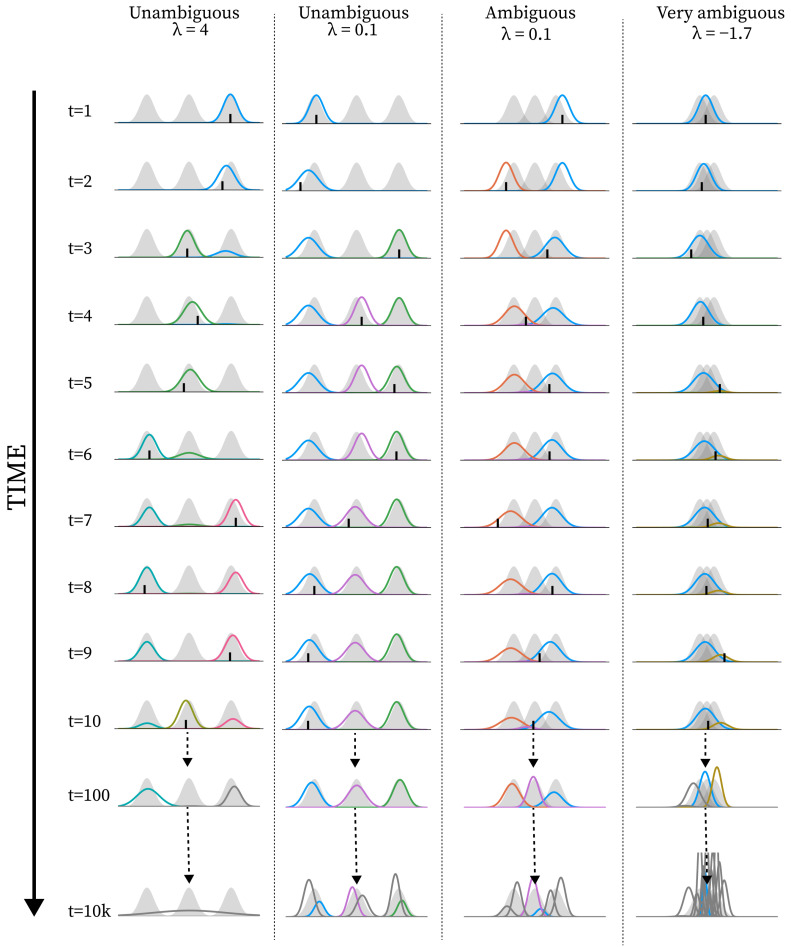
Examples of how the learning dynamic unfolds with different levels of λ at different levels of ambiguity. The plot is constructed in the same way as described in Figure 3, except that there are four columns, with the level of ambiguity and the level of λ used denoted at the top of each column.

In Figure 8, we see examples of how the structure learning dynamic might unfold at various values of λ diverging from the standard parametrisation. Notice here how the over- and under-differentiation of the observation space is similar to that observed for varying σ values in the long-term, but not in the short-term (see Figure 5).

**Figure 9:**
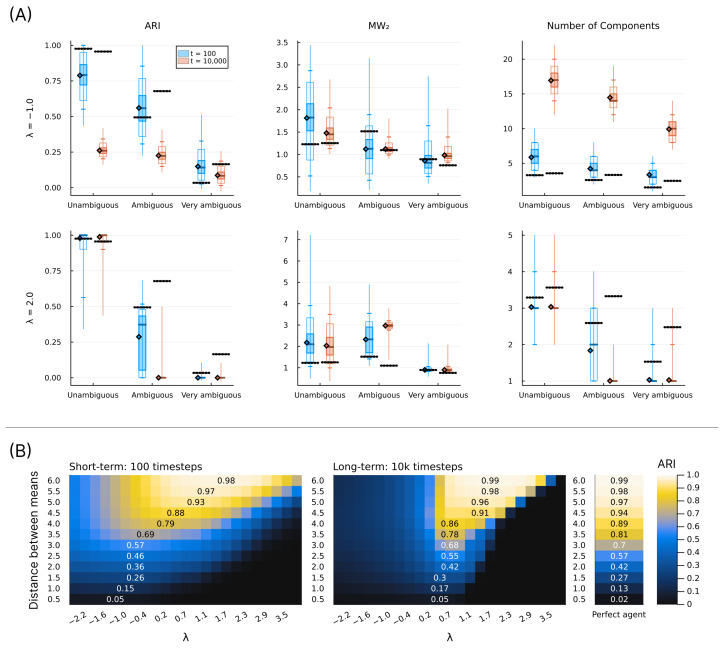
Measures at different values of λ and at different levels of ambiguity. All plots are produced in the same way as in Figure 6, except that varying λ values are used and σ=0 across all simulations.

In Figure 9A, we see the distributions reflecting what is observed in Figure 9B, similar to the equivalent case of σ in Figure 6, taking into account that the values chosen reflect different parameter regions in terms of behavioural optimality. What is interesting to note here is that the number of components is generally much larger in the long-term than in the short-term for a decreased λ value.

In Figure 9B, we see the same general pattern for varying λ values in the short-term as for σ values in Figure 6B. For long-term simulations, λ values lower than the standard parametrisation (λ=0.7) result in worsened performance, and for higher values, there is a sharp decrease in performance at specific values depending on the level of ambiguity.

**Figure 10:**
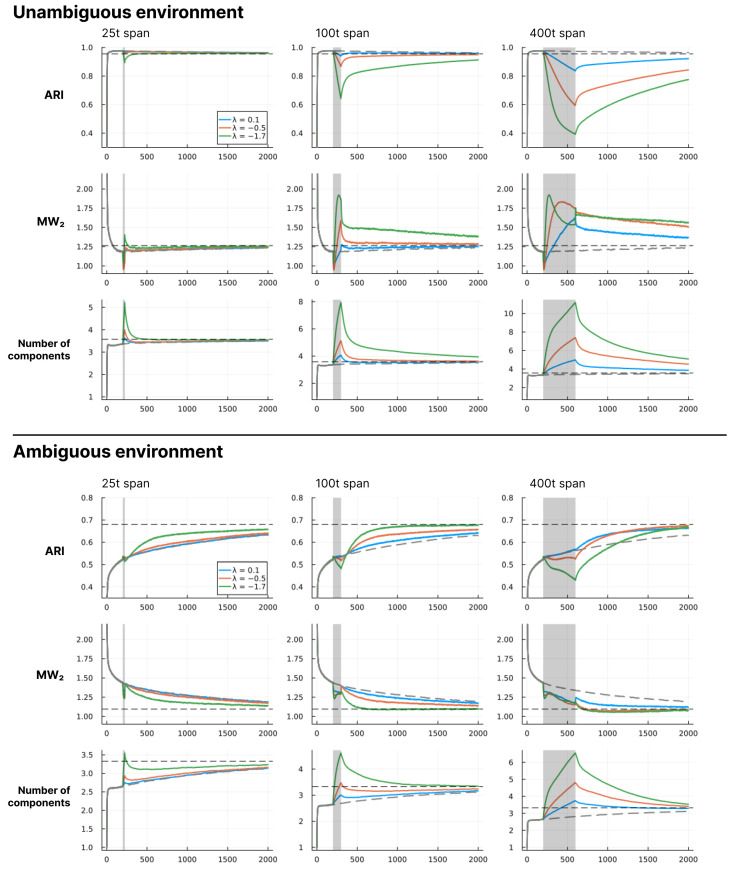
Plots showing the average trajectory of ARI, MW_2_ and number of components across two levels of ambiguity and three spans of altered λ. The figure is produced in the same way as in Figure 7, except that λ is altered with the spans and σ=0 throughout. Blue lines correspond to λ=0.1 within the span, red lines to λ=0.5, and green lines to λ=−1.7. For all simulations, λ=0.7 before *and* after the span.

For ARI in Figure 10, we also see the same general pattern as for σ in Figure 7, with the noticeable difference that the shape of the curves is inverted. Here, the decrease and increase in average performance are more rapid at the start and end of the span, respectively, and the shift is instantaneous at the end of the span. We also see a more rapid decrease in number of components after the span than what was observed for σ in Figure 7. For MW_2_ in Figure 10 we see a spike in improvement at the beginning of the span rapidly followed by an increased distance. In some cases, we observe the reverse at the endpoint of the span, suggesting that changing the λ value may result in some rapid changes to the overall mixture density, perhaps related to a sudden change in the number of components. Within the ambiguous environments we see that the MW_2_ distance decreases on average within the full span, meaning that in this condition the increased number of components better capture the overall density of the environment.

### 3.3. Investigating Interaction Between σ and λ

**Figure 11:**
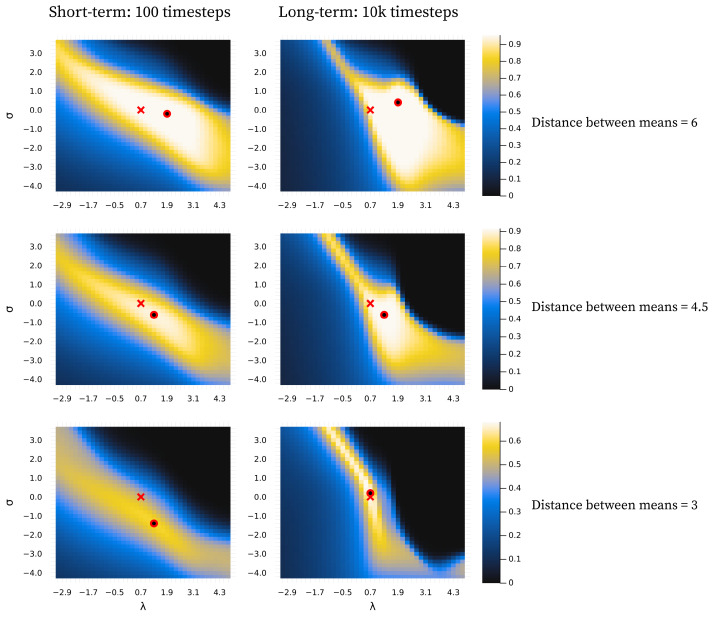
Model performance under varying σ and λ values. Each cell represents average behavioural performance, produced and depicted in the same way as in Figure 6B and Figure 9B. The x-axes represent varying λ values, and the y-axes represent varying σ values. Each row of plots represents a different environment, denoted to the right of the row. Note that each row uses a different mapping to the colour scale, and therefore colours should not be compared across these three rows. The convergence point under standard parametrisation for the given environment (i.e., the average behavioural performance after 100,000 timesteps) is used as the top value of the colour scale. This is done to more clearly depict the shape of the distribution in each plot. The red x marks the standard parametrisation (σ=0.0, λ=0.7), and the red circle marks the cell with the maximum value within each plot.

In Figure 11, we show that there is a negative relationship between σ and λ in terms of optimal behaviour. This means that, to some degree, one parameter can compensate for the behavioural effects produced by the other, with some areas of parameter space being generally better. We also clearly see that the standard parametrisation lies within the preferable range across all conditions shown, except the most ambiguous in the short-term, which is consistent with what we observe elsewhere. We note that similar patterns are observed for all measures (see Appendix A).

The difference between the best behavioural performance of the trained models and the constructed perfect agent (agents with a component structure identical to the environment) in Figure 6B and Figure 9B is very similar across all levels of ambiguity in the long term. Note that here, *perfect* does not refer to perfect classification. In ambiguous environments, this would be possible only by chance and would not suffice as a measure of perfect learning. Instead, it refers to an agent with a set of components where θ exactly corresponds to the environmental components, i.e., an agent that perfectly represents the environment. As such, the results from the perfect agent provide a benchmark, representing the best performance we can expect the model to achieve under the given conditions. Interestingly, in highly ambiguous environments the optimum for the trained models is slightly better than that of the perfect agents. However, performance for both the trained models and the perfect agents is close to chance level. In the short-term, as the environment becomes more ambiguous, the average performance difference increases between the optimum for the trained models and the perfect agents. This is due to a slower trajectory towards the convergence point, as exemplified by the grey lines in Figure 7 and Figure 10.

In Figure 6B and Figure 9B, we see the same general pattern for varying σ and λ values in the short-term. However, this pattern emerges for slightly different reasons. Both show worse performance for lower values in unambiguous environments because too many components are present, i.e., the observation space is over-differentiated. This is partially due to both parameters indirectly affecting how easily new components enter the model. However, in the case of small λ values, the model remains in this state because, as λ decreases, it becomes harder to eliminate inappropriate new components due to increased constraints on weight updating. This is also why we see a large increase in the average number of components in the long-term for the decreased λ in Figure 9A. In the short-term, for ambiguous environments, the lower optimum for λ arises because lower λ values allow for the model to tolerate a higher level of ambiguity, whereas in the case of σ, the lower optimum is due to its ability to differentiate with higher precision. However, as exemplified in Figure 5 and Figure 8, consistently low values of both parameters almost always result in over-differentiation in the long-term.

## 4. Discussion

The simulation results provide a general proof of principle that our model dynamic can perform structure learning in simple environments under the standard parametrisation given above. This capacity to produce properly differentiated internal categories from undifferentiated observations supports the paper’s core claim, namely that representational structure can emerge from constraint-based dynamics. Additionally, we explored several aspects of the model dynamic related to interactions between regions of parameter space and environmental configuration. In this section, we discuss these aspects further and outline the limitations and future directions for this constraint-based approach.

Our central claim is that we model structure learning as an emergent capacity, understood as continued enabling of learning trajectories organised into a representational structure. Here, the system-level is a model of the whole-to-parts constraint involved in this emergence. As the components would accumulate and converge over time absent of such a constraint, it is clear that these trajectories are emergent possibilities under our operational definition. We have also demonstrated that they are organised over time into a representational structure that differentiates the environmental categories, albeit to varying degrees under varying circumstances. The remaining question is then whether the system-level organisation constrains the learning trajectories such that representational structure continually emerges.

While we have shown this continued emergence for some conditions in the form of stabilisation across long-term simulations, the most compelling result is how the component organisation reconfigures after parameter manipulation as shown in Figure 7 and Figure 10. When parameters are manipulated, the number of components increases, the behavioural performance (ARI) decreases, and the mixture distance (MW_2_) increases (with one exception discussed below), which taken together suggest that on average the parameter manipulation causes the organisation to diverge from being representational of the environmental structure, in a sense, sending the component learning trajectories astray. However, once the standard parametrisation is recovered, the average of all measures eventually returns to the baseline. The basic mechanism here is that the tension within the component organisation results in a pruning of the components that are ill organised. This is evidence that the model implements a dynamic that tends towards a representational structure once unfolded in time, much like an attractor state, i.e., a dynamically enabled organisational constraint on the system. This is exactly the emergent capacity.

The activity-level distribution is constructed to be a model of momentary system-level dynamic constraint satisfaction of external and internal factors. The weight update can then be seen as modelling a historic imprint of these constraints. Here, the function *g* (Equation (26)) plays an important role in facilitating the tension dynamics that drives the emergence of structure learning. We emphasise that this may not be a unique way to approach such constraint-based modelling and that the form of the weight update is not strictly derived from a precise constraint-based principle. The weight update should be seen as our attempt to model self-organising dynamics from a Bayesian constraint-based perspective.

The model shows robust behavioural performance (ARI) in unambiguous environments across a range of σ and λ values (Figure 11). In ambiguous environments, the behavioural optimum lies at lower values of σ (Figure 6B) and λ (Figure 9B) for short-term learning, but less so for long-term learning. However, as seen in Figure 7 and Figure 10, a short-duration decrease in either parameter value may aid long-term learning in ambiguous environments by accelerating structure learning overall. This seems to be generally related to the observed increase in the number of components, which can then later be pruned under standard parametrisation.

A decrease in both σ and λ is associated with a risk of representational over-differentiation (for different reasons). In Figure 11, the black area in the top right corner represents a region of parameter space that often results in under-differentiation. Since these parameter values show the worst performance, under-differentiation may lead to more severe behavioural consequences than over-differentiation. This suggests that, in the face of ambiguity, risking over-differentiation can be advantageous, especially when there is a risk of under-differentiation. Extrapolating from these results, one might expect an adaptive structure learning agent to adjust to novel, transforming, or otherwise informationally obscured environments by increasing the level of structural expansion, despite the risk of over-differentiation. However, the consequences of behaviour may of course be highly context-dependent. At least intuitively, one might argue that it is easier to apply the same behaviour to perceptually distinct phenomena than to precisely apply different behaviour to the same perceptual phenomenon. In fact, in order to do the latter, some perceptual differentiation would have to exist at some level. According to this logic, in an ambiguous environment, it may be safer to over-differentiate in order to retain the option of applying distinct behavioural responses.

It is important to note that the behavioural measure (ARI) used in this study captures potential behavioural performance at a given time point, but does not reflect structural coherence across time. Considering Figure 11, it may seem preferable to combine a relatively large λ value with a relatively low σ value in unambiguous environments. However, large λ values often come at the cost of components being replaced more frequently (e.g., see the first column in Figure 8), and low σ values are associated with similar long-term structural incoherence. In fact, in the case of very low σ values it is rare for components added during the short-term phase (first 100 timesteps) to still be present in the model at t=10,000. We find that our standard parametrisation offers a good balance between flexibility and coherence, a compromise that future work should investigate more rigorously.

The negative correlation observed in Figure 11 may cause concern about the model’s utility in experimental settings, as this could reflect that σ and λ are unidentifiable. As discussed throughout the article and reflected in the results, the effect of varying these parameters is quite different overall, despite the shared tendency for over-differentiation at lower values. We provide a simple parameter recovery study in the Appendix for those interested in this aspect (Appendix C).

The MW_2_ distance revealed a perspective not visible in the ARI or component count measures. Most notably, in the ambiguous environment under λ manipulation, the MW_2_ distance decreased during the span of altered λ, despite an increasing number of components (Figure 10). Because environmental components overlap substantially, additional model components can contribute to covering the combined environmental density, reducing the MW_2_ distance regardless of whether they constitute appropriate structural differentiation. This pattern was not observed under σ manipulation (Figure 7), where the added components are highly precise and therefore concentrate density narrowly rather than broadly covering the environmental density.

Under σ manipulation, MW_2_ distance recovered faster than the ARI, suggesting that the narrow components added during the span are drawn towards the environmental density before tension dynamics take sufficient effect for behavioural alignment (Figure 7). Under λ manipulation the recovery pattern is different. Because the added components are less precise, tension between components is more immediately present when parameters shift back, producing a more abrupt reorganisation visible in the sharper decrease in the number of components (Figure 10).

We note that the relationship between the present approach and Bayesian inference accounts deserves some reflection. As discussed above, the activity-level calculation has a formal structure paralleling CRP-based inference, yet the model was not designed with inference as its target. A central difference concerns whether strict categorical assignment is a perceptual or a behavioural act. In DPMM inference, the posterior over component assignments is used to assign each observation to a component during learning, implicitly treating categorisation as part of perception. In the present model, assignment only happens at the response stage, when the representational structure is used to produce a discrete output. This suggests that structure learning may not require direct categorisation at the perceptual level.

The present model may serve as inspiration for researchers interested in incorporating online contraction into DPMM-based frameworks. The most fundamental departure from standard DPMM is the replacement of discrete accumulating counts with a continuous bidirectional weight $w_{i,t}$, where the function *g* plays a central role by providing a structure-independent scaling of relevance that drives tension between components and thereby enables contraction. The role of *I* should also be clarified and rather than a posterior over assignments, $a_{i,t}$ is better understood as a posterior distribution over the inferred component relevance of an observation. A further consideration concerns the role of the component-level processes. Within an inference reading, these could be viewed as replacing the approximation algorithm used to run system-level inference online, but here the HGF components are not approximating the system-level posterior. Alternatively, they could be viewed as sitting at an upper hierarchical level relative to each component at the system-level in the sense of hierarchical Bayesian models. How either of these readings would be formally sustained remains to be clarified, but we hope these pointers are useful for researchers wishing to engage with the model from a more epistemic perspective. Finally, the component weights $w_{i,t}$ do not natively conform to a probability distribution, meaning that the mixture is not a proper Gaussian mixture model until the weights are normalised, which is a practical consideration for any adaptation of our approach.

We have positioned our model interpretation in relation to a Bayesian inference approach that treats inference concretely as a process regarding epistemic entities. However, it may not be the case that all Bayesian inference modellers necessarily subscribe to such a literal interpretation. Some may use the inference framework more as an epistemically worded terminology for the formal syntax, rather than an actual epistemic interpretation. Relaxing the inference interpretation also blurs the differences to our dynamic constraint satisfaction approach. However, we suggest that the difference in framing is not merely terminological. It invites substantially different ways of thinking about the model and more importantly different ways of relating it to biological systems.

In an inference framing, the relationship to biology is usually established by asking whether and how the brain implements or approximates a given computation. In a constraint-based framing the relationship can be approached differently, as the model describes organisational dynamics, and the question becomes whether those dynamics capture something about how biological systems are actually organised. This shift is also motivated by evidence that the brain is an inherently active system [7,8]. If this is the case, the fundamental modelling challenge is not only how the system responds to input, but how ongoing inherent activity is shaped by environmental influence, which is the type of question a constraint-based framing is suited to address. This shift in framing changes what counts as a good model and what kind of evidence is relevant for evaluating it. As such, it could fundamentally change how one would approach biological measurements like neuroimaging data in relation to the model.

Importantly, the formal resemblance between the present model and inference accounts should not be seen as a problem for this framing. On the contrary, it raises the possibility that constraint-based dynamics may provide a grounding for understanding *how* biological systems produce behaviour that is well described by Bayesian inference accounts. The idea is that the constraints organising biological systems give rise to behaviour that conforms to inference-like regularities precisely because they can be described with the same Bayesian logic. We regard this as a promising direction for future theoretical work rather than an established conclusion.

Our results show that, under certain parametrisations (e.g., low σ or low λ), the model develops representations that do not align with any component of the environment and that components can remain relatively fixed over extended time periods, especially for low λ values. This resembles delusion-like phenomena, where false beliefs are fixed and resistant to counter evidence [50,51]. Within our model, such misaligned and relatively fixed representations emerge from constraint-based dynamics under certain parametrisations, offering a possible computational analogue. Here, both low σ and low λ promote the emergence of delusion-like representations through an elevated propensity for structural expansion. The property of being fixed could then be explained by a resistance to tension, which is a direct effect of decreased λ and an indirect effect of overly precise components. The role of α has not been investigated here but warrants attention in future work, as it also controls the baseline propensity for structural expansion.

One aspect the model may shed light on is the gradual and behaviourally silent onset of delusions. As shown in Figure 7, perturbations to the structure learning dynamic can alter learning trajectories with delayed behavioural effects, suggesting that the seeds of representational misalignment can develop before it becomes behaviourally detectable. This is consistent with observations suggesting that delusional beliefs may emerge gradually without a clear behavioural onset [52,53]. While our present results do not directly support claims about delusionality, the model shows properties that provide an interesting direction for the computational modelling of delusions.

This work presents a proof of concept rather than a fully fleshed-out framework for modelling cognition. It has not been our intention to provide a competing model, but rather to explore an alternative approach. For this reason, we have not included an empirical comparison with other modelling frameworks, as this would be beyond the scope of this paper. The comparisons we do provide are at a formal and theoretical level, situating the model within the broader landscape of structure learning.

We explore only two of the four free parameters in the current implementation (three system-level parameters and one component-level parameter). A systematic investigation of interactions across the full parameter space would be too broad for the scope of this paper. Our decision to focus on σ and λ was guided by preliminary analyses suggesting that these parameters reveal particularly informative aspects of the approach. Nevertheless, the full parameter space should be explored in future work.

While we argue for using Bayes’ theorem as a probabilistic expression of constraint, not every constraint playing a role in the dynamic is formally modelled in this way. As is evident from the descriptions of various aspects of the dynamics, some constraints emerge as the system evolves. Furthermore, not every formal aspect of the model is explicitly framed in constraint-based terms. At the component level, we use the HGF framework to model Bayesian inference as a learning process. Although the concept of filtering may intuitively relate to constraints, it remains to be clarified how the component level should be interpreted within a fully constraint-based framework if structure learning is to be understood entirely in these terms. Nonetheless, we aim to show that the model is grounded in Bayesian logic expressing a set of core system-level constraints from which structure learning emerges.

We provide no formal proof or guarantee that the model will converge to a true or optimal structure. The convergence point for unambiguous environments seen in Figure 4 (green box) is slightly lower than the average behavioural performance (ARI) reached in the short-term. However, we still see that at least half of the simulation runs result in perfect categorisation in both short- and long-term simulations. The lowered mean is a consequence of a slightly longer-tailed distribution, reflecting the non-zero probability that unfavourable observation sequences lead to improper structures over time.

The model and simulation setup are intentionally simplistic. The observation space is low-dimensional, the environment is static, and there are no behavioural interactions during learning. Extending the model to incorporate behaviour in a dynamic environment as part of the constraint-based dynamics will be a crucial next step. Given that the model builds on constrained spontaneous activity, here implemented as component-level learning processes, it would be natural to extend this approach to behaviour itself. This could involve treating the cognitive system as a mechanism for filtering appropriate behaviour for an otherwise spontaneously behaving agent, potentially integrating behaviour directly into the representational model via component activity levels. Such an approach contrasts with the common separation of learning and response models, in which perception and action are treated as distinct processes. Instead, perception would be modelled as a capacity emerging from constrained spontaneous action.

Future work should also extend the model to incorporate more abstract representations, such as object classes or generalisations. This could perhaps be achieved by performing structure learning in representational space in addition to what is implemented here. One may argue that due to the lack of abstraction as well as the lack of explicit memory, the model presented here is best understood as a model of very low-level processes. In this way, our model may be a good description of an initial dimensionality reduction of complex environmental information, which serves as a representational basis for other mechanisms of abstractions and explicit memory. Future work should explore whether abstractions and memory require specialised mechanisms, or whether they can arise as emergent capacities within constraint-based dynamics, similar to how we have shown that structure learning can emerge.

The central motivation for this work was to explore whether cognitive systems can be computationally modelled as constraint-based systems. In this respect, our simulation results are promising and provide an initial justification for pursuing this approach. One potential advantage of a constraint-based framework is that it may facilitate translation between cognitive and biological processes by allowing for both to be described using a shared conceptual vocabulary. Such a framework could help us to bridge neurobiology and cognitive science, an essential step towards understanding the relationship between emergent cognitive capacities and measurable neural activity, including the effects of neurobiological interventions on cognition, such as pharmaceutical treatments in psychiatry.

## Figures and Tables

**Figure A1:**
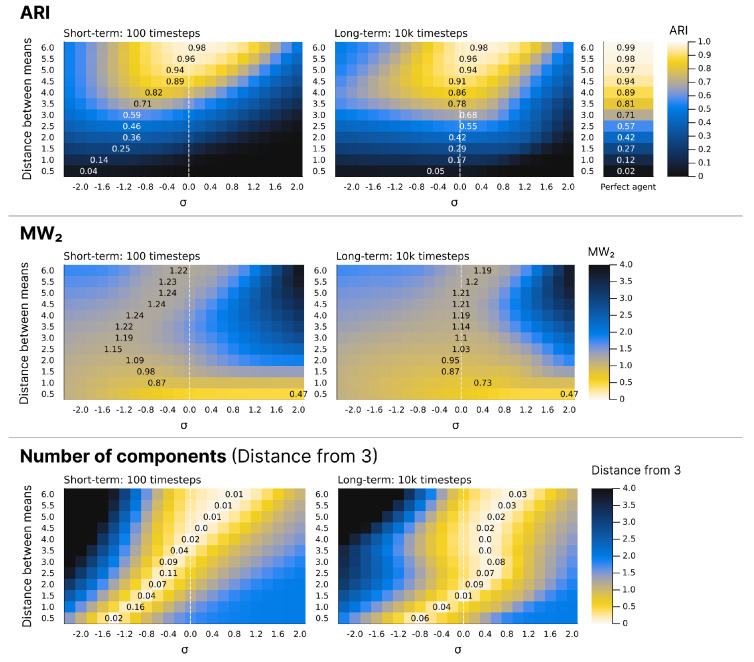
Extra results regarding σ. The top row (ARI) is a copy of Figure 6B. The second and third rows contain the same information, but using the MW_2_ distance or the number of components (measured as the distance from 3).

**Figure A2:**
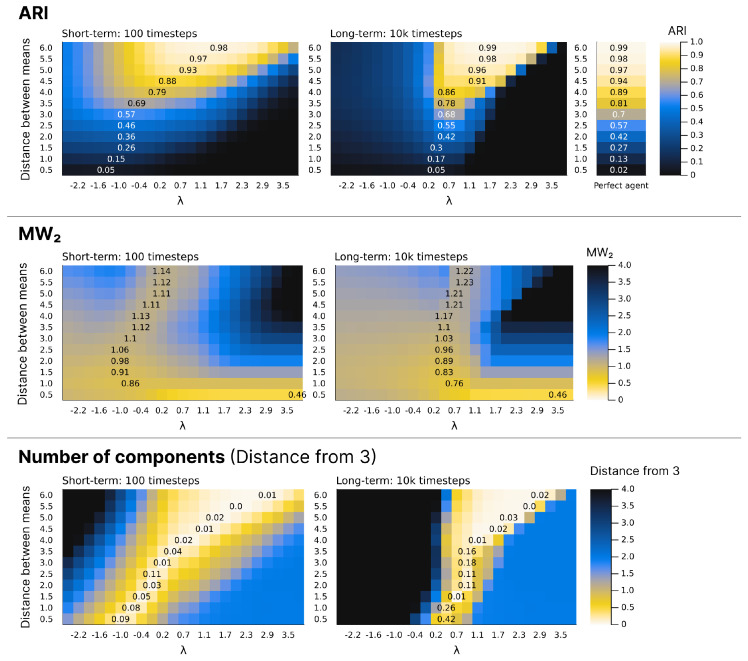
Extra results regarding λ. The top row (ARI) is a copy of Figure 9B. The second and third rows contains the same information, but using the MW_2_ distance or the number of components (measured as the distance from 3).

**Figure A3:**
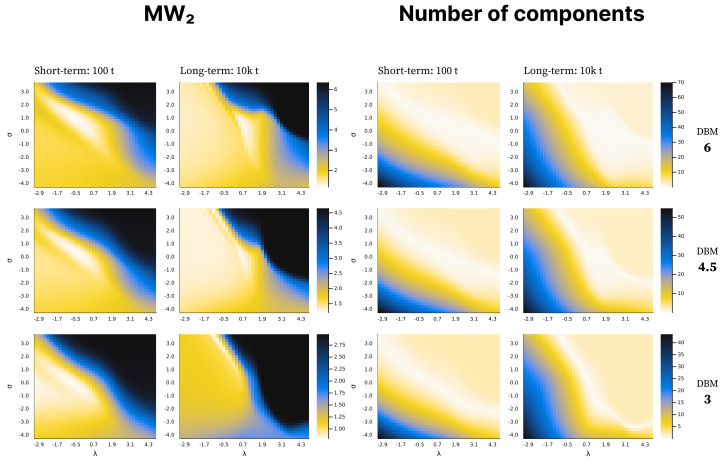
Extra results regarding the interaction of σ and λ. The two plots mirrors Figure 11, but using MW_2_ distance or the number of components (measured as the distance from 3) instead of ARI.

**Figure A4:**
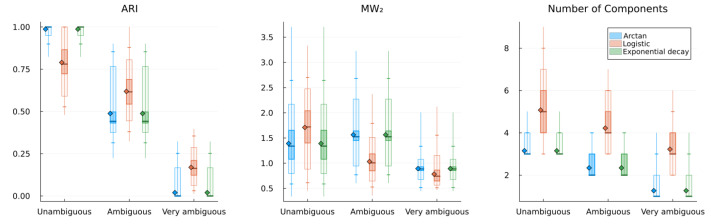
Distributions of measures after 100,000 timesteps using the standard parametrisation described in the main text. The plots are constructed the same way as Figure 4 in the main text, except that the colours represent the different mapping functions used.

**Figure A5:**
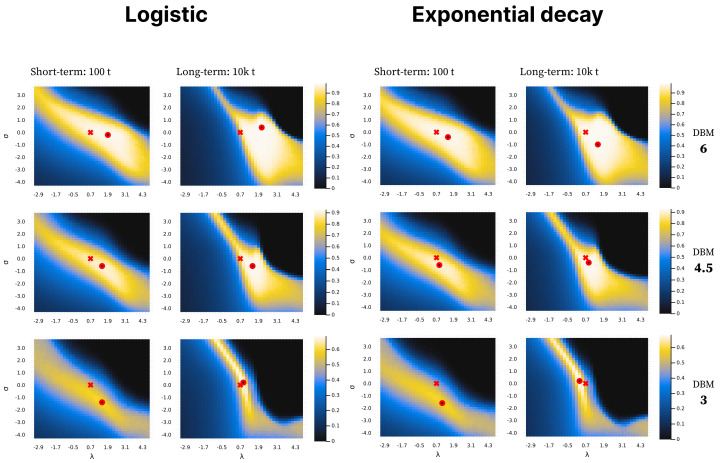
Interaction of σ and λ using the logistic (**left**) and exponential decay (**right**) mapping functions. In addition to using different mapping functions, the plots are constructed the same as Figure 11.

**Figure A6:**
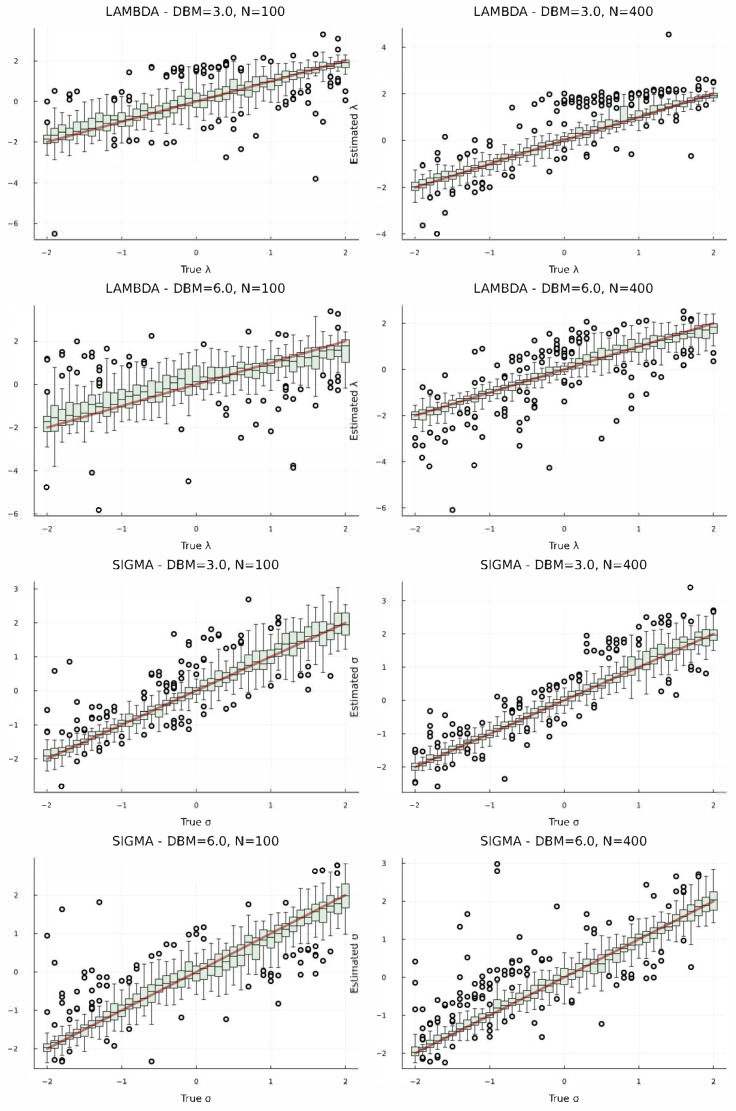
Each panel shows the distributions of recovered posterior medians (boxplots) plotted against the true generating parameter values (x-axis), with the diagonal line indicating the x=y relationship. Boxes summarise the distribution of estimates across the full grid of the other parameter. Columns separate the two parameters (left: λ; right: σ). Rows correspond to the four conditions crossing environment ambiguity (DBM denoting distance between means) with session length (N = 100 vs. N = 400 observations). Dots represent outlier values. Recovery is good where the boxplots track the diagonal line closely.

## Data Availability

All scripts and data used for the production and analysis of the results are available at https://doi.org/10.17605/OSF.IO/RMVGB (accessed on 30 January 2026).

## Supplementary Materials

The following supporting information can be downloaded at https://www.mdpi.com/article/10.3390/e28050534/s1. For all animation files (.gif), six models are shown. Within the same file, all models receive the same sequence of observations, but they differ in their parametrisation. It is noted above each model animation how it differs from the standard parametrisation. Here, the colour of the components reflects the time during the simulation at which they were added. The colour gradient at the bottom shows the progression of colours across timesteps. The timestep of each frame is also shown. Note that for long-term animations and trajectory animations, only every 10th and 5th update is shown, respectively, to speed up the total animation time. Trajectory animations are intended as examples of the trajectory plots in the article. Here, the background changes to grey during periods of alternate parametrisation. The parameter shown at the top of each simulation denotes the parametrisation used during that time span. When the background is white in the trajectory animations, all models use the standard parametrisation. The file names indicate the content of the animations. Here, DBM denotes “distance between means”. DBM6 corresponds to an unambiguous environment, and DBM3 to an ambiguous environment.

## Appendix B. Alternative Choices For The Arctan Function

The arctan function used to implement *g* (Equation (26)) is not the only possible mapping into a probability space. Here, we assess the robustness of the main results to alternative functional forms. Specifically, we compare the arctan function used in the main text to a logistic function and an exponential decay function adapted to map onto a probability space for positive input values. Let $\phi =\frac{e^{\lambda }}{{\left(o_{t}-\mu_{i,t}\right)}^{2}\tau_{i,t}}$ define the input to the mapping functions. The three functions tested are

(A1) $$\mathrm{Arctan}\left(\phi \right)=\frac{2}{\pi }\arctan\left(\phi \right)$$

(A2) $$\mathrm{Logistic}\left(\phi \right)=\frac{1-e^{-\phi }}{1+e^{-\phi }}$$

(A3) $$\mathrm{Decay}\left(\phi \right)=1-e^{-\phi }$$

As shown in Figure A5, the key findings are largely preserved across all three functional forms, with the main difference being that the choice of function slightly shifts the parameter space for λ in terms of optimality. Figure A4 reflects this in the differences observed at the standard parametrisation used in this paper. These results suggest that the core model behaviour is not critically dependent on the specific choice of function used to implement *g*.

## Appendix C. Parameter Recovery Study

To assess whether σ and λ can in principle be identified from behavioural data, we ran a parameter recovery study using the ActionModels.jl package (https://computationalpsychiatry.github.io/ActionModels.jl/stable/, accessed on 30 January 2026). For each combination of true σ and λ on a grid spanning [−2, 2] in steps of 0.1, we simulated an agent producing categorical reports for observations drawn from a three-component Gaussian environment and then fit the same model to the simulated data using Metropolis–Hastings sampling (2000 samples) under weakly informative priors (λ∼N(0.7,2), σ∼N(0,2)). The posterior median was taken as the point estimate. We repeated this across two environments: unambiguous (distance between means = 6) and ambiguous (distance between means = 3). For each environment, we tested two session lengths (100 and 400 observations). Figure A6 shows the recovered estimates against the true values. The results suggest that both parameters can be recovered above chance level across all conditions, supporting the conclusion that σ and λ are in principle identifiable, consistent with them governing distinct aspects of the model dynamic.
