# Supplementary figures and images for: It Is What It Isn’t: Introducing a Constraint-Based Approach to Structure Learning
Source: Entropy (Basel). 2026 May 7;28(5):534. doi: 10.3390/e28050534 (PMC13206405; doi:10.3390/e28050534)

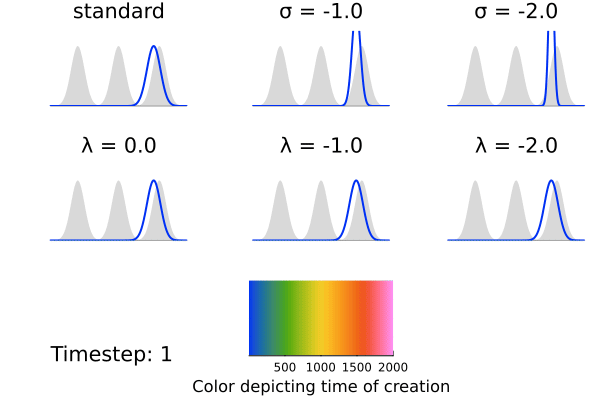

Supplement: Supplementary file 1 [file entropy-28-00534-s001.zip › animations/long-term_DBM6.gif]

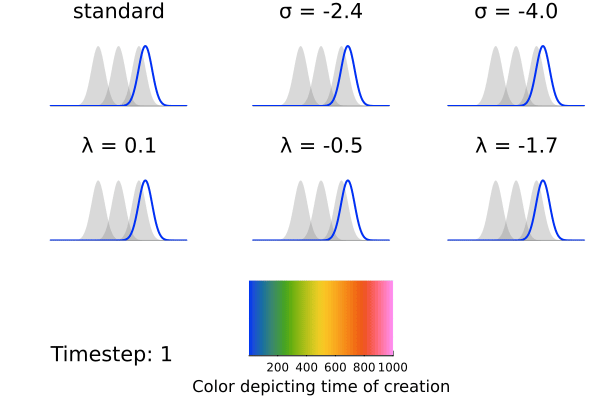

Supplement: Supplementary file 1 [file entropy-28-00534-s001.zip › animations/trejactory_DBM3_span100.gif]

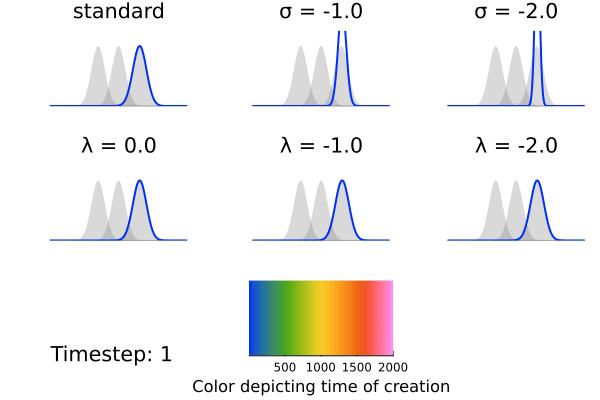

Supplement: Supplementary file 1 [file entropy-28-00534-s001.zip › animations/long-term_DBM3.gif]

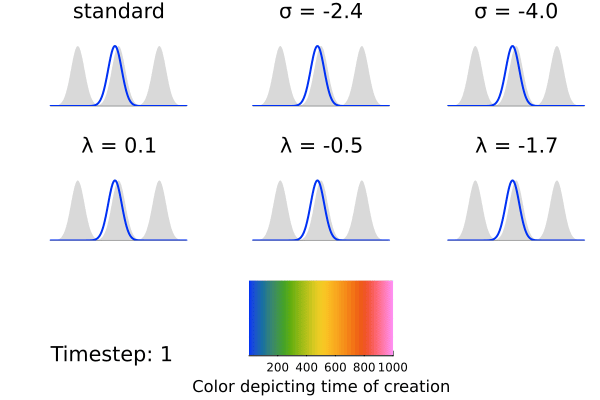

Supplement: Supplementary file 1 [file entropy-28-00534-s001.zip › animations/trejactory_DBM6_span100.gif]

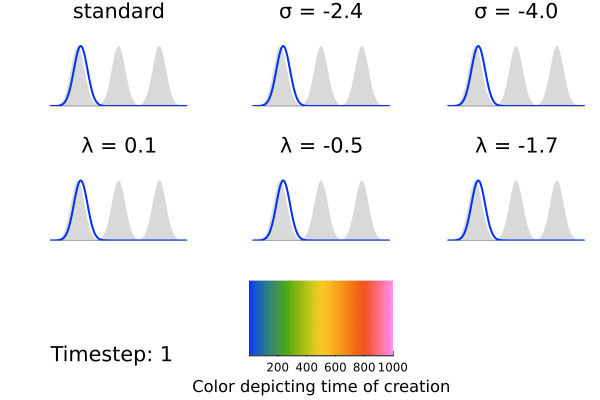

Supplement: Supplementary file 1 [file entropy-28-00534-s001.zip › animations/trejactory_DBM6_span400.gif]

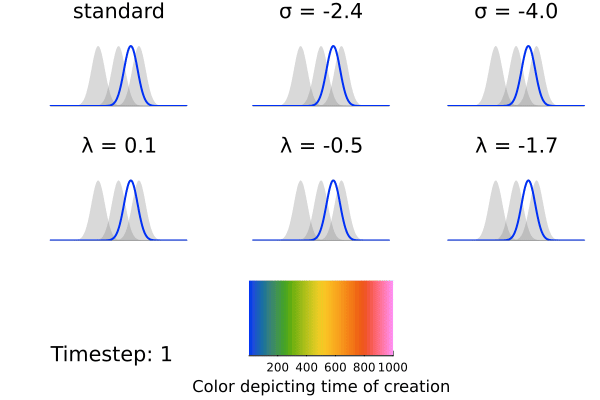

Supplement: Supplementary file 1 [file entropy-28-00534-s001.zip › animations/trejactory_DBM3_span400.gif]

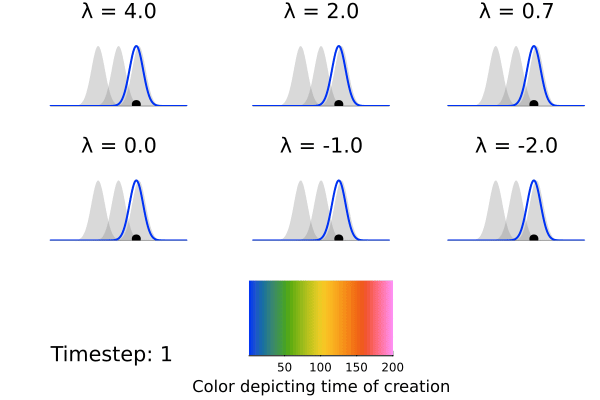

Supplement: Supplementary file 1 [file entropy-28-00534-s001.zip › animations/lambda_DBM3.gif]

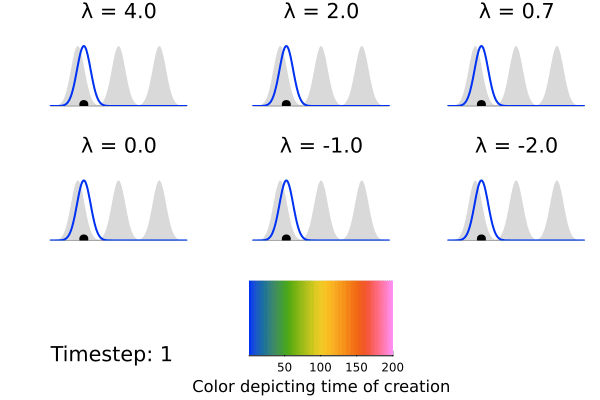

Supplement: Supplementary file 1 [file entropy-28-00534-s001.zip › animations/lambda_DBM6.gif]

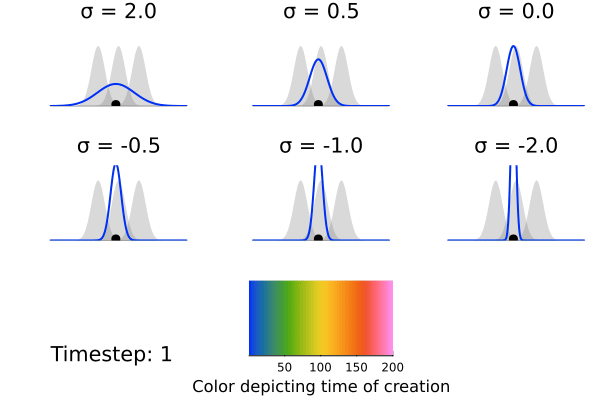

Supplement: Supplementary file 1 [file entropy-28-00534-s001.zip › animations/sigma_DBM3.gif]

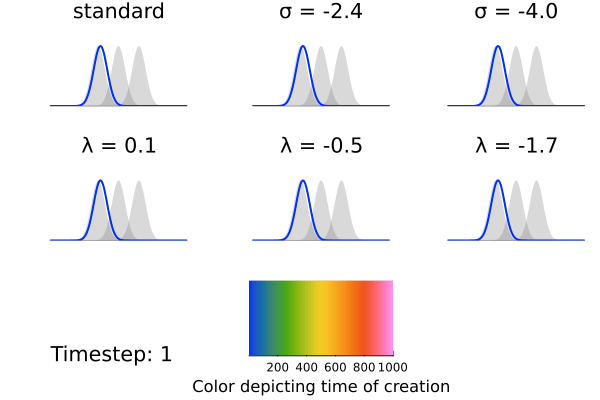

Supplement: Supplementary file 1 [file entropy-28-00534-s001.zip › animations/trejactory_DBM3_span25.gif]

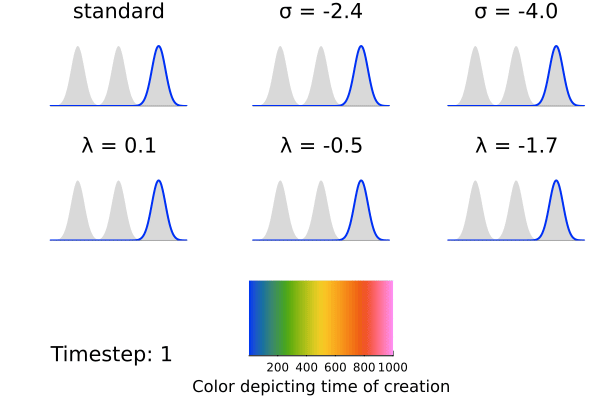

Supplement: Supplementary file 1 [file entropy-28-00534-s001.zip › animations/trejactory_DBM6_span25.gif]

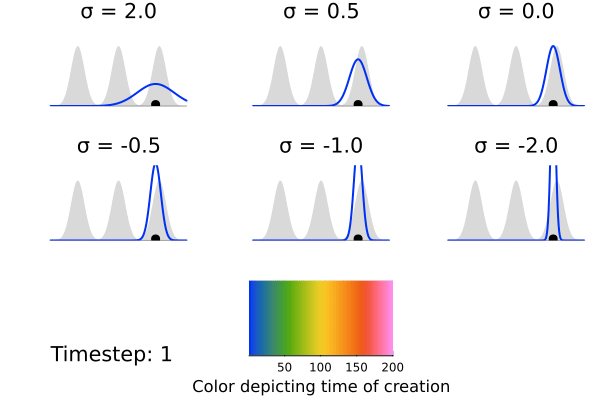

Supplement: Supplementary file 1 [file entropy-28-00534-s001.zip › animations/sigma_DBM6.gif]
